# Dysregulated Signalling Pathways Driving Anticancer Drug Resistance

**DOI:** 10.3390/ijms241512222

**Published:** 2023-07-30

**Authors:** Nauf Bou Antoun, Athina-Myrto Chioni

**Affiliations:** School of Life Sciences Pharmacy and Chemistry, Biomolecular Sciences Department, Kingston University London, Kingston-upon-Thames KT1 2EE, UK; k1741364@kingston.ac.uk

**Keywords:** cancer, tumourigenesis, drug resistance, signalling pathways, Wnt/β-catenin pathway, JAK/STAT pathway, PI3K/Akt/mTOR pathway, RAS/RAF/MAPK/ERK signalling

## Abstract

One of the leading causes of death worldwide, in both men and women, is cancer. Despite the significant development in therapeutic strategies, the inevitable emergence of drug resistance limits the success and impedes the curative outcome. Intrinsic and acquired resistance are common mechanisms responsible for cancer relapse. Several factors crucially regulate tumourigenesis and resistance, including physical barriers, tumour microenvironment (TME), heterogeneity, genetic and epigenetic alterations, the immune system, tumour burden, growth kinetics and undruggable targets. Moreover, transforming growth factor-beta (TGF-β), Notch, epidermal growth factor receptor (EGFR), integrin-extracellular matrix (ECM), nuclear factor kappa-light-chain-enhancer of activated B cells (NF-κB), phosphoinositol-3-kinase/protein kinase B/mammalian target of rapamycin (PI3K/Akt/mTOR), wingless-related integration site (Wnt/β-catenin), Janus kinase/signal transducers and activators of transcription (JAK/STAT) and RAS/RAF/mitogen-activated protein kinase (MAPK) signalling pathways are some of the key players that have a pivotal role in drug resistance mechanisms. To guide future cancer treatments and improve results, a deeper comprehension of drug resistance pathways is necessary. This review covers both intrinsic and acquired resistance and gives a comprehensive overview of recent research on mechanisms that enable cancer cells to bypass barriers put up by treatments, and, like “satellite navigation”, find alternative routes by which to carry on their “journey” to cancer progression.

## 1. Introduction

Over many years, cancer has consistently been a significant global public health burden. According to GLOBOCAN, in 2020, there were estimated around 19.3 million new cancer cases and 10.0 million cancer-related deaths (all cancers combined excluding non-melanoma skin cancer) worldwide [1,2]. Cancer continues to be the greatest cause of mortality in the world despite the enormous progress that has been made in developing more effective treatment options, partially due to drug resistance that is challenging to bypass. The heterogeneous, versatile and adaptable nature of cancer to overcome “obstacles” put up by treatments are some of the key reasons why it has proven difficult to combat drug resistance, and therefore, effectively and significantly reduce relapse and mortality.

Carcinomas can be thought of as organ-like structures made up of both transformed cancer cells and nontransformed stroma [3,4]. A tumour stroma is a complex milieu and a crucial component of the tumour microenvironment (TME), which is highly active, heterogeneous and frequently tumour-type specific. It consists of noncellular components such as the extracellular matrix (ECM; including secreted exosomes, metabolites, chemokines and cytokines) and the distinctive vascular system associated with cancer as well as a wide range of cellular elements such as activated cancer-associated fibroblasts (CAFs), pericytes and mesenchymal stromal cells (MSCs). It also includes immune cells (such as natural killer (NK) cells, T and B lymphocytes and tumour-associated macrophages (TAMs)) and lymphatics [5,6,7].

Although there are many different types of disorders that are placed under the umbrella term “cancer”, the formation of abnormal cells that proliferate beyond their natural bounds serves as both a defining characteristic and a common denominator. Normal cells gradually transition into the neoplastic stage because tumour formation is a multi-step process, and along the way, they gain specific abilities that make them tumourigenic. These fundamental signature traits, which are both unique and supplementary, include continuing proliferative signalling, eluding growth inhibitors, allowing replicative immortality, avoiding cell death, generating angiogenesis, reprogramming of energy metabolism, avoiding immune destruction and triggering invasion and metastasis as well as the relative autonomy of cancer cells in relation to the stroma. These traits are characterised by genomic instability, which constructs the genetic variety that expedites their inflammation and acquisition, which supports many hallmark functions. These distinguishing traits and enabling qualities describe essential components for cellular transformation [8,9].

Throughout the past few decades, chemotherapy, radiation, hormone therapy, targeted therapy and immunotherapy have all undergone remarkable advancements in their development and therapeutic use for cancer [10]. However, the occurrence of intrinsic and acquired resistance to treatments in cancer patients has been a significant barrier that limits the efficacy of cancer treatments and has an impact on patient survival [11,12]. A complete understanding of the interactions between tumour cells and their microenvironment is required to fully comprehend the acquirement of growth advantage and drug resistance in tumour cells. This review aims to address the complexity of interactions within a tumour, recent findings on distinct drug resistance signalling pathways and strategies for combating anticancer drug resistance and enhancing its effectiveness.

## 2. Evolution of Cancer

Depending on the type of tumour, where it is located and what stage of disease it is in, the TME’s composition and structure change. The following criteria are used to categorise the stages: (i) development of the primary tumour; (ii) invasion of cancer cells into nearby tissue; (iii) spread of cancer cells to distant organs via the blood or lymphatic system; (iv) extravasation into a secondary organ stroma; and (v) development of secondary tumours and metastasis [12,13].

**Figure 1:**
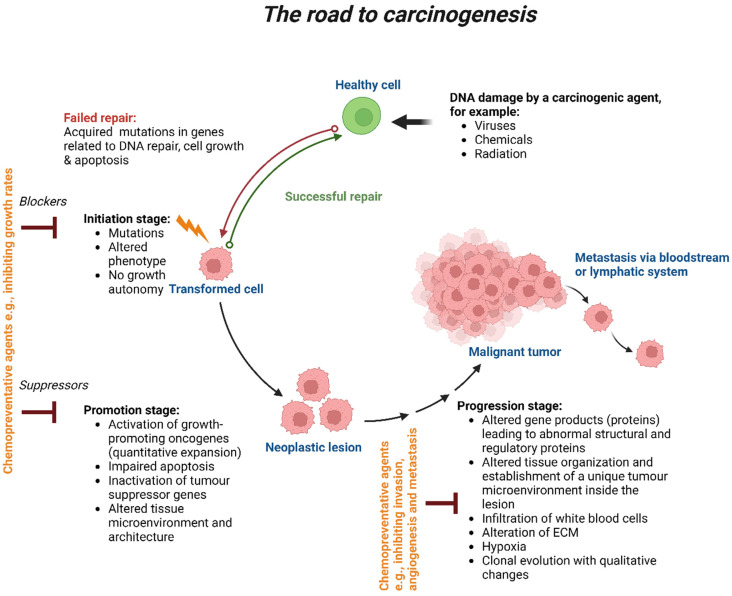
Stages of carcinogenesis. Exposure to a carcinogenic agent, such as viruses, chemicals or radiation, will induce DNA damage in one or a small population of healthy cells. Failure in DNA repair will lead to intrinsic or acquired mutations that will potentially affect the cell’s biological process, such as its growth and apoptosis, resulting in initiating premalignancy transformation. The transformed cells will then promote the growth of neoplastic lesions, which hold so many genetic alterations. Chemopreventive agents, such as natural agents derived from dietary sources (e.g., curcumin, resveratrol) or bioactive molecules (tamoxifen, raloxifene), will be used to block and suppress cell growth rates at those stages to reverse or delay the carcinogenesis process. Failure to eliminate those premalignant cells will lead to the progression stage, resulting in the formation of a malignant tumour. Chemopreventive agents will be given at this stage to inhibit cell invasion, metastasis and angiogenesis. Failure to do so will result in metastasis via the bloodstream or lymphatic system. Created with BioRender.com.

The four stages of carcinogenesis are initiation, promotion, progression and metastasis (Figure 1). Initiation is the first stage in which the mutation of genes can naturally occur or because of carcinogen exposure. The molecular signalling pathways involved in cellular proliferation, differentiation and survival can become dysregulated due to genetic alterations. Numerous factors, including the type and rate of carcinogenic metabolism as well as the response of the DNA repair function, have an impact on these pathways. During the protracted and reversible promotion stage, a build-up of actively proliferating preneoplastic cells takes place. Chemopreventive medicines may alter the mechanism at this point and impact growth rates. The transitional period between the emergence of a premalignant lesion and the beginning of an invasive malignancy is referred to as progression. This latter is the last stage of neoplastic transformation, which involves genetic and phenotypic alterations as well as cell proliferation. This entails a sharp increase in tumour size and the possibility of new mutations in the cells, which could lead to invasion and metastatic dissemination. Chemopreventive medications should have the ability to work more effectively at the stages of carcinogenesis initiation and promotion. Cancer cells spread to different parts of the body through the process of metastasis, which involves the lymphatic or circulatory systems. Chemopreventive drugs have been shown to prevent angiogenesis and the invasion of primary tumours; as a result, they may be used to limit cancer metastasis [14].

## 3. Drug Resistance in Cancer

**Figure 2:**
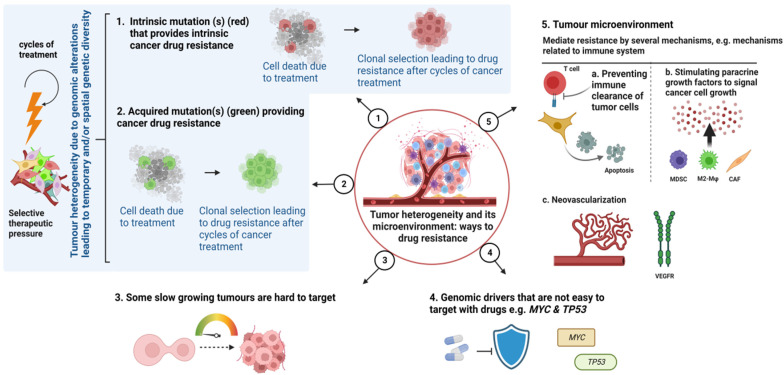
Potential mechanisms in cancer drug resistance. Several mechanisms trigger drug resistance in cancer. Exposure of cancer cells to therapeutic pressure induces genomic alterations and mutations, either (1) intrinsic, represented in red, or (2) acquired, represented in green, after cycles of treatment, which in both cases result in drug resistance. (3) Slow-growing tumours and (4) intractable genomic drivers (e.g., MYC and TP53) play a critical role in the emergence of drug resistance. All these factors play a vital role in tumour heterogeneity leading to genetic diversity. (5) Tumour microenvironments mediate resistance by several mechanisms, e.g., (a) escaping immune surveillance, (b) stimulating paracrine growth factors by tumour-associated cells to promote cancer cell growth and (c) the neovascularization of tumour cells by overexpressing vascular endothelial growth factor receptors (VEGFR). All these factors make a complex network that triggers drug resistance in cancer. Created with BioRender.com.

Cancer therapy, in its most basic form, consists of a treatment that works against a group of cancer cells located in a particular host environment. The range of clinical responses is caused by the pharmacological qualities of the therapy, as well as the intrinsic and acquired molecular and physical features of cancer cells and external environmental factors [15]. Many studies have focused on the differences between intrinsic and acquired cancer drug resistance; however, a variety of overlapping mechanisms can also be contributing factors. By comprehending the underpinnings of resistance, to both current and upcoming treatments, we can develop a paradigm of mechanisms that takes into consideration all variables of cancer and therapeutic science that can influence it (Figure 2).

Therefore, the development of drug resistance is a complex phenomenon that involves several different components and numerous interconnected signalling pathways. Altered drug targets, modified drug metabolism, enhanced drug efflux, repair of DNA damage, epigenetics modifications, dormancy, undruggable targets, TME and epithelial-mesenchymal transition (EMT) are some of the fundamental molecular mechanisms by which cancer cells develop chemoresistance. The molecular mechanisms linked to drug resistance in cancer are explained below, and a schematic sketch is shown in Figure 2.

### 3.1. Intrinsic Drug Resistance

The natural resistance that occurs before the patient is exposed to medications is typically referred to as intrinsic resistance and can influence treatment effectiveness. The latter can be brought on by several factors, such as increased DNA repair capacity, altered drug metabolism, mutated or altered drug targets, reduced drug accumulation and deactivated cell death signals [11,12]. 

Cancer stem cells (CSCs) exhibit drug resistance because they overexpress adenosine triphosphate (ATP)-binding cassette (ABC) transporters [16]. Through certain regulatory genes, FOXM1, a transcription factor specific to cell proliferation, controls the transition between the G1/S and G2/M cell cycle phases. Additionally, it is an oncogene that promotes the expansion and multiplication of cancer cells [17]. Through the expression of ABCC5 (ATP binding cassette subfamily C member 5), FOXM1 overexpression causes paclitaxel resistance in nasopharyngeal carcinoma [18]. Growth differentiation factor-15 (GDF-15) is a member of the superfamily of transforming growth factor-beta (TGF-β). Proliferation, angiogenesis, stemness, metastasis, drug resistance and immunological modulation are all associated with the overexpression of GDF-15 in cancer. It was demonstrated that stemness and indicators of treatment resistance were significantly positively correlated with GDF-15 expression in breast cancer patients. This suggests that the p-Akt/FOXM1 axis mediates the relationship between increased GDF-15 expression and enhanced stemness and treatment resistance in breast cancer [19]. Oestrogen receptor positive (ER+)/ human epidermal growth factor receptor 2 positive (HER2+) breast cancer is strongly influenced by the HER2-E subtype and erbB2, which results in resistance to endocrine therapy and a higher probability of recurrence [20]. About 20–30% of metastatic breast tumours overexpress the human epidermal growth factor receptor 2 (HER2/erbB2), which is associated with a poor prognosis [21]. In studies using trastuzumab as the sole treatment, over two-thirds of patients showed intrinsic resistance to the drug [22,23]. High levels of GDF15 may be a factor in trastuzumab resistance in HER2 overexpressing breast cancer cells through the activation of TGF-β receptor-Src-HER2 signalling crosstalk [24]. Furthermore, the aberrant activation of the phosphoinositol-3-kinase/protein kinase B/mammalian target of rapamycin (PI3K/Akt/mTOR) signalling pathway is closely related to resistance to anti-HER2 treatment [25]. Moreover, due to the genetic mutation(s) of genes involved in cancer cell proliferation and/or death, intrinsic drug resistance may develop in cancer cells before therapy. For instance, HER2 overexpression induced EMT and promoted resistance to cisplatin in gastric cancer cells [26]. CSCs and EMT are both associated with intrinsic drug resistance via these concurrent alterations mentioned above [27,28].

Intercellular genetic heterogeneity in cancer can result from genomic instability, which is characterised by mutations, gene amplifications, chromosomal rearrangements, gene deletions, gene translocations and alterations in microRNA [29]. Moreover, genotypic changes can have an impact on epigenetic variables affecting the heterogeneity of the mRNA, transcriptome and proteome [30]. 

### 3.2. Acquired Drug Resistance

Gradual decline in a drug’s ability to treat cancer after treatment can indicate acquired resistance. A number of factors can contribute to acquired resistance, including changes in the TME following therapy through various mechanisms, such as low pH, hypoxia, shifts and polarisations in the immune cell population, exosomes, various secretomes, vascular abnormalities and soluble factors derived from stromal cells [11,12,31]. Paracrine signalling connections between stromal and tumour cells, mutations or altered levels of drug target expression and activation of a second proto-oncogene that develops into the driver gene, can also contribute to acquired drug resistance [11,12,32].

Targeted medicines cause subtler alterations that can be classified as acquired resistance after repeated exposures or early adaptive responses. Adaptive responses may be the cause of transient clinical reactions because they might happen so quickly that no response is ever clinically evident. Adaptive processes are frequently the result of epigenetic modification and/or non-genetic relief of negative feedback of signalling pathways, which activates parallel pathways or reactivates the initial one [33,34]. For example, due to the reactivation of upstream receptor tyrosine kinases (RTKs), such as epidermal growth factor receptor (EGFR), BRAF-mutant colorectal tumours are resistant to BRAF inhibitors, while low levels of EGFR expression in BRAF-mutant melanomas were not affected by the negative feedback relief [35,36].

New genetic mutations can cause resistance and regeneration in cancers that had previously shrunk. Whole-genome sequencing comparing the genetic profiles of eight patients with acute myeloid leukaemia before and after relapse revealed novel gene mutations (e.g., DAXX, DDX41, DIS3, SMC3 and WAC) responsible for tumour resistance and regeneration [37]. Chemotherapeutic medications disrupt the DNA of malignant cells, which probably accelerates the occurrence of new mutations. In addition, also linked to acquired chemoresistance is the crosstalk that occurs between tumour cells and their microenvironment as the disease progresses [38]. This will be discussed later in the TME section below (Section 3.10). 

Some non-small-cell lung cancer (NSCLC) patients experience acquired resistance due to circumstances that can interfere with EGFR signalling, such as the upregulation of other RTKs such as MET, the downstream activation of specific pathway elements or phenotypic and histological changes [39]. Recently, it was demonstrated that EGFR signalling pathways were activated by autocrine EGF and TGF-α and withstood c-Met and anaplastic lymphoma kinase (ALK) inhibition leading to primary and acquired resistance to TAE684/SGX-523 (ALK/c-Met inhibitors) in NSCLC [40]. In hepatocellular carcinoma (HCC) cells that heavily express c-MET, hepatocyte growth factor (HGF) activated the downstream PI3K/Akt and mitogen-activated protein kinase/extracellular signal-regulated kinase (MAPK/ERK) pathways through c-MET and concurrently reduced the anticancer effects of lenvatinib (a tyrosine kinase inhibitor) and promoted EMT [41]. Activating PIK3CA mutations in HER2+ breast cancer will unable a favourable response to pyrotinib plus trastuzumab neoadjuvant therapy [42]. By encouraging FOXD1 translation through PIK3CA/PI3K/Akt/mTOR signalling, FOXD1-AS1 (an oncogenic long non-coding RNA (lncRNA)) exacerbates gastric cancer development and chemoresistance [43]. Moreover, one of the primary causes of medication resistance is the point mutation in the c-ros oncogene 1 (ROS1) gene. ROS1 is a receptor of the insulin family of tyrosine kinases. Recently, it was demonstrated that the point mutations CD74-ROS1 D2033N and CD74-ROS1 S1986F render NSCLC cells resistant to crizotinib via FAK/PI3K/Akt signalling pathway activation [44].

The activation of hypoxia-inducible pathways, EMT, the interaction between the PI3K/Akt and Janus kinase/signal transducers and activators of transcription (JAK/STAT) pathways and the enrichment of tumour-initiating cell population are some of the processes that cause acquired resistance to sorafenib [45]. Furthermore, potential mechanisms have been revealed that underlie acquired resistance to gemcitabine in gallbladder cancer, such as the disruption of drug metabolism and the activation of receptor and non RTK (i.e., PDGFRA, ABL1 and LYN), as well as the increased expression of EMT-related markers, FN1, CDA and LAMC2 [46].

A significant increase was observed in single-nucleotide variants in the genes ATM, ATR, BRCA1, LRP1B, MAP2K1, PIK3CG and ZNF217 in addition to BRAF, KRAS, NRAS and EGFR among tumours receiving prior anti-EGFR [47]. Genes with possible signalling implications and those involved in DNA repair pathways made up the two main categories of these changes. ZNF217 and PIK3CG converge on Akt1 signalling, which may encourage anti-EGFR-acquired resistance. Protein kinase MAP2K1, the acquisition of which occurs following anti-EGFR therapy, enhances the translation of signalling from MEK to ERK [48,49]. LRP1B inhibits β-catenin signalling [50]. β-catenin activation might be a different route by which to get around EGFR suppression, similar to Akt1 bypass signalling. It is interesting to note that after exposure to anti-EGFR, a greater frequency of mutations in BRCA1, ATR and ATM was found. These modifications could aid in the evolution of a response to targeted therapy and could also account for increases in relative tumour mutation burden (rTMB) in patients exposed to anti-EGFR leading to acquired resistance [47].

The activation of EGF like domain multiple 7 (EGFL7)/Notch signalling in lung cancer cells, triggers resistance to EGFR inhibitors [51]. Furthermore, SNHG7 (a lncRNA) activates the Notch1/Jagged1/HES1 pathway resulting in tumour cell stemness and resistance to folfirinox in pancreatic cancer cells [52].

### 3.3. Altered Drug Targets

One of the main causes of drug resistance is drug targeting alteration, which occurs when drug targets’ expression and functionality are altered. Di(2-ethylhexyl) phthalate (DEHP) is a chemical that is frequently found in everyday items and polyvinylchloride medical equipment. As a result, phthalates can enter the human body through eating, inhalation and medical procedures. Phthalates induce cancer progression and chemotherapeutic resistance [53]. Recently, it was demonstrated that DEHP increased trefoil factor 3 (TFF3) expression through the vinculin/aryl hydrocarbon receptor (AhR)/ERK signalling pathway, which induced EMT [54]. In human breast cancer, the expression of the oncogene TFF3 is favourably connected with both ER+ and negative cells, and it increases cell metastasis, invasion, proliferation and treatment resistance [55]. Through the ubiquitination pathway, DEHP promoted AhR-related changes in oestrogen receptor expression, which reduced tamoxifen’s effects in AhR knockout mice [54].

A T315I point mutation that arises in the BCR-ABL kinase domain is the most frequent mutation in BCR-ABL that causes resistance to first-generation (imatinib) or second-generation tyrosine kinase inhibitors (TKIs) that target the BCR-ABL protein, leading to a poor clinical prognosis in chronic myeloid leukaemia [56,57]. Due to the activation of intrinsic signalling pathways, such as the RAS/RAF/MAPK/ERK, GSK3 β and JAK/STAT5 pathways, imatinib intolerance or initial resistance arises, and many leukaemic patients acquire secondary resistance [58,59,60,61]. Most cellular intrinsic mechanisms play a role in the development of resistance, either directly through BCR-ABL1 point mutations, which predominate in primary resistance, or indirectly through the activation of signalling pathways independent of BCR-ABL1, which frequently lead to disease recurrence and therapy relapse [59,62]. Typically, such activation frequently occurs in a BCR-ABL1-independent manner; as a result, those oncogenic pathways continue to be active even after treating leukaemic cells with imatinib, including nonmutated BCR-ABL1 cells.

Moreover, osimertinib is a third-generation powerful EGFR-TKI used to treat NSCLC patients with EGFR mutations. The therapeutic use of osimertinib is nonetheless restricted by the emergence of acquired resistance associated with the triple mutation Del19/T790M/C797S in EGFR [63]. Furthermore, mutations at either V550 (a gatekeeper residue) or C552 (hinge-1 residue) in the kinase domain of fibroblast growth factor receptor 4 (FGFR4) prevent fisogatinib (a potent and selective FGFR4 inhibitor) from interacting with the FGFR4’s ATP binding site, resulting in acquired clinical resistance to fisogatinib in patients with HCC [64].

TKIs can prevent downstream pathways from being improperly activated by aberrant protein tyrosine kinases (PTKs). PI3K/Akt, RAS/MAPK/ERK and JAK/STAT are examples of key signalling pathways that control a variety of cellular processes by stimulating proliferation, encouraging angiogenesis, preventing apoptosis and promoting drug resistance [65]. Therefore, due to mutations at the drug binding sites, TKIs lose the ability to inhibit PTKs (e.g., FGFRs, EGFRs, ALK, platelet-derived growth factor receptor (PDGFRs), insulin-like growth factor receptor (IGFRs), vascular endothelial growth factor receptor (VEGFRs)), resulting in constant activation of downstream signalling pathways.

### 3.4. Modified Drug Metabolism

Once ingested, drugs are biochemically transformed by drug metabolism enzymes. Metabolic activation is necessary for many anticancer drugs to carry out their mechanism of action. These enzymes have been linked to drug activation and inactivation in cancer cells, including the uridine diphosphoglucuronosyltransferase (UGT) superfamily, glutathione S-transferase (GST) superfamily and cytochrome P450 (CYP) system [66,67]. A modification in CYP may alter the capacity of these proteins for drug metabolism, leading to both a large increase in drug release and a change in how the drug is broken down. As a result, patient intratumoural medication concentrations drop, and the treatment loses its effectiveness. Recently, it has been shown that docetaxel’s resistance in HCC cells may be significantly mediated by the metabolic deactivation of the CYP isoforms 3A4 (CYP3A4) [68].

Key metabolic enzymes of 5-fluorouracil (5-FU) include thymidylate synthase (TYMS); 5-FU is a chemotherapy drug and TYMS is one of its target enzymes. It was established that SNHG15 (lncRNA) increased 5-FU chemoresistance in colorectal cancer (CRC) by controlling TYMS expression [69]. Moreover, the activation of detoxification systems that serve as a defence against contaminants can limit the therapeutic efficiency of anticancer medicines. A compromised detoxification mechanism in cancer cells makes medication responses inefficient and encourages resistance. One of the primary contributing causes to the development of treatment resistance in cancer is the exclusion of medicines by GST [70]. Numerous biological activities, including cell differentiation, proliferation and death, depend on GST. Drug resistance is influenced by an increase in glutathione. For instance, the upregulation of GST-π production and activation of the PI3K/Akt/mTOR signalling pathway were promoted by regenerating gene 4 (REG4) overexpression, which contributed to an invasive phenotype and induced cisplatin and paclitaxel resistance in ovarian cancer. [71]. Furthermore, by blocking the MAPK pathway, GSTs contribute to the emergence of drug resistance [70].

### 3.5. Enhanced Drug Efflux

The term “drug efflux” refers to the rise in the efflux of cytotoxic medications by active ATP-binding cassette (ABC) transporter proteins, which is one mechanism of drug resistance. Chemotherapy can only be successfully used to a limited extent because of these drug efflux transporters, which lower intracellular drug concentration and inhibit therapeutic response [72,73,74,75]. Humans have been found to have 48 members of the ABC transporter family. There are only 13 different types of ABC transporters that have been found to play a part in drug resistance in cancer (ABCC1/2/3/4/5/6/10, ABCB1/2/5, ABCA2/3 and ABCG2) [76]. Breast cancer resistance protein (BCRP/ABCG2), P-glycoprotein (P-gp/MDR1/ABCB1) and multidrug resistance-associated protein 1 (MRP1/ABCC1) are three major ABC transporters that have recently undergone substantial research to better understand how multiple drug resistance (MDR) works [77,78].

The role of proteins and signalling pathways in the regulation of ABC transporters in cancer cells has been extensively documented in recent years. Through interactions with upstream and downstream targets, the PI3K/Akt pathway, which is elevated in many human malignancies, has been found to be a critical elusive link in MDR. This signalling pathway promotes the progression of cancer and confers resistance to chemotherapy treatments by increasing the expression of the ABC transporters BCRP, MRP1 and P-gp [79,80].

In the human acute lymphoblastic leukaemia cell lines, activation of the MAPK/ERK and JNK pathways upregulated the expression of the ABCB1 and ABCG2 genes, respectively [81]. Research has also revealed that the ABCG2-mediated multidrug resistance in colon cancer cells is caused by the JNK1/c-jun signalling pathway [82]. Interestingly, it was recently found that activation of the RhoB/PI3K/Akt pathway-mediated overexpression of ABC transporters by hsa-miR-3178 is an intriguing mechanism that promotes gemcitabine resistance in pancreatic cancer cells [83]. 

Through the SIRT1/CREB/ABCG2 signalling pathway, miR-132 has also been shown to increase cisplatin resistance in LGR5+ gastric cancer stem-cell-like cells [84]. ABC transporter proteins are highly expressed on the cell surfaces of CSCs, which have been found to have a significant role in drug resistance and play a role as indicators for CSC isolation and identification [85,86,87,88,89,90]. Furthermore, LGR5 is a receptor that enhances the wingless-related integration site (Wnt)/β-catenin signalling pathway, promoting the growth and self-renewal of CSCs. It was recently discovered that a strong cooperation between the expression of LGR5 and LRP6 (mediators of Wnt/β-catenin signalling) was enhanced in neuroblastoma-resistant cells [91]. The higher β-catenin expression in those neuroblastoma cell lines with acquired resistance to vincristine or doxorubicin indicates β-catenin-dependent Wnt signalling [91]. Moreover, a correlation between elevated nuclear factor kappa-light-chain-enhancer of activated B cells (NF-κB) activation/phosphorylation and upregulated MDR efflux transporter expression was demonstrated in doxorubicin-resistant breast cancer cells [92].

### 3.6. Repair of DNA Damage

Numerous chemotherapeutic drugs heavily focus on targeting DNA [93]. Resistance to drugs that target DNA, however, might be brought on by increased DNA repair and tolerance to DNA damage [94]. By causing DNA damage, several chemotherapy medicines, including 5-FU and cisplatin, destroy cancer cells. Drug resistance may occur from DNA lesion repairs caused by the DNA damage response of damaged cells to anticancer medications [95]. The upregulation of p53-target genes on DNA damage response and repair was brought about by 5-FU therapy. When the damage was successfully repaired, the resistant colon cancer cell lines experienced decreased cell cycle arrest and apoptosis, more so than the wild-type ones [96]. Moreover, it was discovered that 5-FU-resistant human colon cancer cell lines have increased levels of the DNA repair genes FANCG, FEN1 and RAD23B [96,97].

Checkpoint kinase 1 (CHK1) plays a vital role in DNA damage and response and is a crucial effector in the control of replication. The mechanism of resistance to prexasertib, a CHK1 inhibitor, was investigated in sarcoma xenografts [98]. BCL-xL, an anti-apoptotic protein, was found in higher concentrations and the PI3K and MAPK signalling pathways were phosphorylated more frequently in prexasertib-resistant tumours. Akt, MEK1/2 and ERK1/2 were found to be substantially active in resistant tumours [98]. Other cell lines have also shown increased RAS/MEK/ERK activity in response to CHK1 inhibition [99,100]. Combining prexasertib with MAPK or PI3K inhibitors was not enough to overcome developed resistance in sarcoma xenografts [98]. The stimulation of RAS/MAPK and, to a lesser extent, PI3K/Akt/mTOR signalling by EGFR overexpression or activation promotes cell proliferation and may avoid the replication stress caused by prexasertib. About 50% of triple-negative breast cancer (TNBC) show overexpression of the EGFR, which is linked to poor overall survival [101,102,103]. It was suggested that EGFR activation or overexpression is a factor in the innate resistance of TNBC to prexasertib and may also be responsible for the drug’s low clinical efficacy [104].

### 3.7. Epigenetics Modifications

Cell destiny and pathogenic provenience are greatly influenced by epigenetics. It appears that non-genetic heterogeneity contributes to the development of cancer-causing cells and/or resistance to treatment. Impairment in gene expression is caused by epigenetic alterations, which last for several cell divisions and finally result in non-genetic heterogeneity and treatment resistance [105]. The development of chemoresistance in cancer is fuelled by epigenetic changes that are linked to histone modification, DNA methylation, chromatin remodelling and changes associated with non-coding RNA (ncRNAs) [106]. Accumulating evidence shows that epigenetic changes contribute to the development of various resistance mechanisms, such as improved DNA repair, enhanced drug efflux and defective apoptosis. For example, the chromodomain helicase DNA-binding protein 4 (CHD4), which modulates chromatin remodelling, specifically causes drug resistance in breast cancer gene1/2 (BRCA1/2) deficient cells through aiding DNA damage repair [107]. Recently, it was demonstrated that by interacting with major vault protein (MVP), CHD4 encourages gastric cancer cell proliferation and chemoresistance. As well stimulating drug efflux, CHD4 promotes the reduction in the intracellular concentration of cisplatin. It also enhances the protein interaction between ERK1/2 and MEK1/2 leading to the activation of the MVP/MEK/ERK signalling axis [108].

Moreover, DNA methylation and gene expression profiles of fulvestrant- and tamoxifen-resistant MCF7 derivatives with oestrogen-responsive MCF7 human breast cancer cells were analysed. Resistance to tamoxifen is developed by significant alterations in downstream ER target gene networks, whereas acquired resistance to fulvestrant revealed a general upregulation of growth-stimulatory pathways, including cytokines and cytokine receptors, the EGFR, ErbB2 and related proteins, the Notch pathway, the Wnt/β-catenin pathway and the interferons (IFN) signalling pathway/IFN-inducible genes, were among the prominently altered pathways in MCF7 cells resistant to fulvestrant [109]. For instance, demethylation of DNA near an oncogene’s promoter region would increase the gene’s expression, leading to treatment resistance. In a resistant HCC cell line, thymosin β 4 (Tβ4), a G-actin monomer binding protein, was shown to be enhanced through DNA demethylation and the active modification of histone H3 at the promoter region [110]. In the HCC cell line, overexpression of Tβ4 caused stem cell-like capabilities to develop, as well as in vivo resistance to the VEGFR inhibitor sorafenib [110].

One study demonstrated that sorafenib resistance develops because of the histone demethylase KDM1A, also known as Lysine demethylase 1A (LSD1). They found that cells resistant to sorafenib (a TKI) had a higher capacity for self-renewal. KDM1A’s importance for the stemness of liver CSCs by epigenetic alteration was previously discovered by the same team [111]. They found a potential mechanism by which KDM1A causes resistance to sorafenib through the control of important β-catenin signalling pathway antagonists. They also showed that KDM1A is necessary for maintaining the stemness of resistant HCC cells to sorafenib [111].

The first histone modification enzyme shown to be linked to drug resistance to several anticancer drugs is a lysine demethylase called Lysine demethylase 5A (KDM5A) [112,113]. Erlotinib (an EGFR inhibitor) was less effective against breast cancer cells with amplifications of the KDM5A gene due to the increased expression of a set of genes associated with apoptosis/cell cycle, including the apoptosis effector BCL2 antagonist/killer1 (BAK1) and the tumour suppressor p21 [112]. Lewis lung carcinoma and renal cell carcinoma become resistant to sunitinib (an RTK inhibitor) due to KDM5C, which was discovered to be a significant epigenetic regulator in this process. In patients with NSCLC, KDM1A is crucial for inducing gefitinib resistance by the development of hypoxia through generating EMT [113].

Moreover, by changing gene expression and the structure of regulatory proteins, N6-methyladenosine (m6A), a particular type of RNA alteration, influences the development of tumours [114]. KIAA1429 plays a vital role in m6A methylation or controls ncRNAs, including microRNAs (miRNAs) and lncRNA, to promote the growth and metastasis of many cancers [115,116]. Recently it was demonstrated that the activation of the JNK/MAPK signalling pathway results in m6A KIAA1429-mediated gefitinib resistance in lung adenocarcinoma cells [117].

The effects of mucin 17 (MUC17) on the epigenome of EGFR-TKI-acquired drug resistance was examined in NSCLC cells. Gefitinib/osimertinib-resistant (GR/OR) cells were found to increase genome-wide DNA hypermethylation, notably in 5-UTR related to several oncogenic pathways, where GR/OR cells had a pro-oncogenic effect by decreasing MUC17 expression. The downregulation of MUC17 caused by acquired GR/OR was triggered by a methylation promoter dependent on the DNA methyltransferases1/Ubiquitin-like containing the PHD Ring Finger 1 (DNMT1/UHRF1) complex, which in turn stimulated NF-κB activity [118].

### 3.8. Slow Growing Cells

Tumour cells may have transcriptional plasticity, due to epigenetic reprogramming, which will change them into persister cells. These “persisters” are a collection of cells that are slowly growing and have the potential to either re-grow when therapy is stopped or develop enduring resistance. KDM5B, a member of the KDM5A family, designates a small subset of slow-cycling cells in melanomas that are necessary for ongoing tumour maintenance and are dynamically triggered depending on the microenvironmental situation. These KDM5B-positive cells slowly cycle and have increased self-renewal. They are intrinsically resistant to many cytotoxic therapies, and through a dysfunctional Jagged 1/Notch 1-signalling pathway, they can produce offspring that are extremely proliferative [119].

Recent research has demonstrated that the abnormal expression of nerve growth factor receptor (NGFR), SRY-Box transcription factor 2 (SOX2), AXL RTK and melanocyte-inducing transcription factor (MITF) in melanoma cells make them more susceptible to shift into a persister state in response to RAF and MAPK inhibition [120,121].

In response to targeted kinase inhibitors, the histone H3 lysine 27 trimethylation (H3K27me3)-specific demethylases, KDM6A/B, are activated and crucial for the transformation of naive glioblastoma stem cells into the slow-cycling drug-tolerant persisters (DTPs). Pervasive acetylation (H3K27ac) of cis-regulatory components occurs in conjunction with the transition to the persister state and is made possible by a widespread redistribution of the repressive mark H3K27me3. These persisting cells display primitive neurodevelopmental hallmarks because of this modified chromatin state and heavily rely on Notch signalling [122]. Sharma et al. consistently identified a small fraction of reversibly “drug-tolerant” cells while simulating the acute response to several anticancer drugs in drug-sensitive human tumour cell lines. These cells exhibit a >100-fold decrease in drug sensitivity and continue to exist due to activation of the insulin-like growth factor 1 receptor (IGF-1R) signalling and a modified chromatin state that needs the histone demethylase RBP2/KDM5A/Jarid1A. Individual cells within the population transiently acquire this drug-tolerant phenotype at low frequency, suggesting that drug tolerance is dynamically regulated by phenotypic heterogeneity [123]. In addition, KDM5A is necessary to create a transient chromatin state in EGFR-mutant lung cancer cell lines with elevated expression driven by the IGF-1 signalling pathway in both DTPs and drug-tolerant expanded persisters (DTEPs). This will mediate the development of EGFR inhibitor resistance [123].

The irreversible stop of cell growth known as cellular senescence is what causes tumour-suppressive pathways regulated by p16 and/or p53 to be activated. As a tumour suppressor, the protein p16^INK4a^ (also known as p16) inhibits the activity of cyclin-dependent kinases (CDKs) and slows cell division by delaying the transition from the G1 to the S phases of the cell cycle [124]. Both endogenous and external factors can promote cellular senescence. The three main factors are the shortening of telomere, increased mitogenic signalling created by oncogene activation and non-telomeric DNA damage brought on by chemotherapeutic medicines. Senescence can begin, for instance, when chemotherapy drugs such as doxorubicin and cisplatin cause cell death [125,126,127]. In part, via inhibiting apoptosis, p53 and INK4a/ARF mutations encourage carcinogenesis and treatment resistance [126]. Drug resistance and tumour progression/recurrence have been linked to a mechanism known as an escape from therapy-induced senescence (TIS) [128]. The ability of cancer cells with TIS to acquire stem-cell characteristics explains how they can avoid senescence and relapse [129,130].

Moreover, metastasis, chemoresistance and cancer recurrence are all influenced by tumour dormancy. CSCs frequently exist in a quiescent state where they might stay in the G0/G1 stage and proliferate at a slow rate [131,132]. Quiescence (reversible cell cycle arrest) features help CSCs develop resistance to radiation and chemotherapy because most traditional chemotherapeutic agents target proliferating cells [87,133,134]. For example, the majority of 5-FU-resistant gastric cancer cells with CSC characteristics were quiescent cells that stayed in the G0/1 phase [135]. In response to chemotherapies, CSCs enter quiescence by initiating a complex array of intracellular molecular and epigenetic programmes [131]. The three signalling pathways most frequently engaged in CSC quiescence are Notch, Wnt and p38-MAPK. Active p38 mitogen-activated protein kinase 1 (MAPK1) can cause CSC to enter a dormant state in prostate cancer [136]. It is noteworthy that the Wnt canonical pathway component c-Myc can speed up the CSC cell cycle and encourage CSC reawakening, whereas their inactivation has been directly linked to the onset of reversible quiescence [137,138,139,140].

### 3.9. Undruggable Targets

Several of the most powerful oncogenes and tumour suppressor genes, such as MYC, RAS and TP53, remain intractable despite increasing progress in efforts to target oncogenic driver mutations. Ras proteins were discovered to be oncogenes in the early 1980s, but despite extensive research over more than three decades to identify particular inhibitors, they were thought to be unreachable targets.

In up to 90% of human melanoma, mutated BRAF or mutated NRAS hyperactivate the kinase ERK, according to the examination of genetic changes [141,142]. The rationale for developing targeted inhibitors of mutant BRAF and MEK, the kinase that functions downstream of BRAF to activate ERK, as treatments for advanced melanoma was supplied by these findings [143]. The overall survival of patients significantly increased as a result of the introduction of targeted medicines (MAPK pathway inhibitors such as BRAF and MEK inhibitors) and immunotherapies (immune checkpoint inhibitors). However, a lack of clinical effects, side effects and rapidly escalating treatment resistance limit the long-term efficacy of such treatments. This resistant phenotype is supported by several molecular pathways [144]. Moreover, resistance may also be caused by target indifference, in which the effects of focusing on an oncogenic driver are mitigated by changes either concurrently made to the pathway or later on. This is demonstrated by the fact that resistance to anti-EGFR therapy in colon cancer can be caused by downstream mutations that activate NRAS or KRAS [145]. Recently, it has been shown that JAK/STAT pathway activation occurs as BRAFV600E thyroid cancer cells become resistant to BRAF inhibitors [146]. Interestingly, insensitivity to the inhibition of the MAPK/ERK pathway in advanced melanoma tumours harbouring the BRAFV600E mutation resulted from the activation of compensatory signalling cascades. Particularly in mesenchymal-like cells, the PI3K/Akt/mTOR axis displayed increased activity, resulting in a decreased MAPK/ERK signalling dependency and promoting stem-like features, making the latter pathway’s inhibitors ineffective [147]. 

Cancer frequently harbours mutations in the p53 pathway. In fact, the TP53 gene exhibits mutations or deletions in around 50% of human malignancies, which predominantly cause decreased tumour suppressor activity [148]. Damaged cells may multiply after losing their p53 functioning, passing on changes to the following generation [149]. Deregulation of p53 frequently causes tumour development and mutant p53 cancers are frequently characterised by genomic instability, promoting proliferation, migration, invasion, angiogenesis and increased drug resistance [149,150].

In NSCLC, mutated p53 increases binding to the nuclear factor erythroid 2–related factor 2 (Nrf2) promoter, supported by an activation of the NF-κB signalling pathway, which further increases Nrf2 expression. Nrf2 is a transcription factor that codes for detoxification enzymes and confers resistance to anticancer drugs. In addition, in p53 mutant colon cancer cells, the absence of DNA mismatch repair triggers resistance to cisplatin [151]. Furthermore, mutant p53 affects the ERK-mediated transcription of early growth response-1 (Egr-1) and the ERK pathway, which enhance the production of EGFR ligands and stimulates EGFR signalling, rendering therapy to the EGFR inhibitor ineffective [152]. Moreover, mutations in PI3K and MAPK pathways are common in metastatic CRC and accelerate tumour growth in conjunction with other prevalent mutations in the p53, TGF-β and Wnt signalling pathways [153]. Mutations in the MAPK pathway are present in these CRC patients (0.8% in MAP2K1, 1.7% in MAP2K4, 3.9% in NRAS, 8.5% in BRAF and 44% in KRAS). The PI3K/Akt/mTOR pathway is mutated in CRC patients (1% in AKT1, 2.4% in PIK3R1, 2.5% in PIK3CG, 2.8% in PTEN and 18% in PIK3CA) [5]. Additionally, 11% of the remaining patients exhibit mutations in RTKs, which are upstream of both pathways triggering the emergence of resistance mechanisms to chemotherapy or targeted therapies [15,154].

Breast, colorectal, liver and other cancers are all mostly driven by the MYC oncogene. More than 70% of human malignancies exhibit high and/or abnormal Myc expression, which is associated with aggressive diseases and a bad prognosis [155,156]. Myc is a difficult oncoprotein to target due to its high frequency of overexpression in malignancies and its pervasive function in transcriptional control. There are presently no specific medications that can be used to target Myc, primarily due to its “undruggable” characteristics: Myc is primarily localised in the nucleus, making it inaccessible to antibodies and lacks an enzyme site where typical small molecules can bind [157]. BRD4 is a crucial epigenetic regulator (a chromatin regulator) and a member of the BET family. The human genome contains regulatory components, including silencers (repressors), enhancers/super-enhancers and promoters, that are used to dynamically modulate the regulation of transcription. In BET inhibitor-sensitive leukaemia cells, the classic enhancer or super-enhancer controls MYC expression through BRD4 binding. The expression of MYC is inhibited and cell proliferation is suppressed as a result of the BET inhibitor’s blocking of BRD4’s ability to bind to its genomic targets. However, through various mechanisms, long-term drug therapy may restore MYC expression. One of those mechanisms is maintaining MYC expression by activating Wnt/β-catenin signalling pathways, which results in enhanced β-catenin binding to the sites that were initially occupied by BRD4, leading to drug resistance [158].

The assessment of tumour heterogeneity is a crucial clinical concern. Genomic sequencing is used to assess heterogeneity in cancer samples that were either archived at the time of diagnosis or were later biopsied upon recurrence. This method has significant limitations because it is unlikely to adequately capture tumour heterogeneity, which has clear consequences for cancer therapy despite its utility in some circumstances for therapy selection [159]. Targeting an ‘actionable’ driver mutation, for instance, might only be successful if the mutation is truncal (i.e., clonal and present in the majority of subclones and parts of the tumour during the course of its lifetime) [160]. In other situations, the presence of a particular mutation may not indicate that it is clonal, and vice versa, the scarcity of a mutation does not indicate that it is accidental. In fact, resistance to targeted medications can be brought on by subclonal driver mutations in the PI3K pathway genes and ESR1. A list of the ‘clonality’ of driver mutations might be helpful in this case [161,162].

### 3.10. Tumour Microenvironment

Cancer cells, stromal cells, ECM, blood and lymphatic vessels, immune cells, nerve fibres, signalling molecules and related acellular components make up the TME. The latter is sculpted and instructed by cancer cells to support the emergence of cancer hallmarks, react to stimulation, internal or external stress and therapy and eventually support the survival, growth, angiogenesis, migration, invasion and immune evasion as well as drug resistance of these cells [10].

TME consists of myeloid-derived suppressor cells (MDSCs), mast cells, CAFs, TAMs, vascular endothelial cells, adipocytes, pericytes, tumour-associated neutrophils, dendritic cells and granulocytes. It also includes malignant cells, NK cells and T and B cells. Cancer is protected from immunological eradication by the suppressive immune microenvironment [4,163]. Regulatory T (Treg) cells, neutrophils, macrophages, MDSCs, CD4^+^, FOXP3^+^ and CD25^+^ assist in establishing an immunosuppressive pre-metastatic microenvironment [164,165]. The activation of MDSCs, TAMs and CAFs by reactive oxygen species (ROS) was demonstrated to be crucial in strengthening their immunosuppressive functions [166,167]. Immune cell recruitment into the TME can be affected by the ECM. For example, the ECM can activate the pro-survival pathway PI3K/Akt, which makes it easier for CSCs to evade the immune system [168]. The recruitment of immunosuppressive cells such as Tregs and TAMs by ECM proteins has also been demonstrated to support CSC survival while inhibiting the recruitment of cytotoxic T cells, which are anti-tumourigenic immune cells [169,170,171]. Moreover, lipid metabolism has been associated with tumour progression, recurrence and exhaustion of CD8 T cells through the activation of programmed-cell death protein-1 (PD-1), which results in escaping the immune surveillance after treatment [172,173].

Key aspects that define cancer stemness, the recruitment of non-malignant cells that support tumour cells and ECM remodelling are coordinated by cellular crosstalk via several signalling networks, such as the juxtracrine and paracrine pathways [174]. The suppression or modification of interferon-gamma (IFN-γ) signalling, activation of the MAPK and Wnt/β-catenin pathways, a decreased T-cell response and tumour antigen production are a few often found pathways that inhibit the immunotherapy response leading to treatment resistance [175].

Avoiding detection and eradication by the immune system results in multidrug resistance [176]. PD-1 is frequently expressed on the membranes of immune cells such as macrophages and T and B cells. While various tumour cells express programmed death ligand 1 (PD-L1). It has been demonstrated that the interaction of PD-1 and PD-L1 on T cell surfaces can inhibit the activity of killer T cells by promoting apoptosis, which causes tumour cells to escape the immune system [177]. Through the IL-6/STAT3/PD-L1 axis, CAFs modulated neutrophil activation, survival and function in tumour tissues in HCC to promote immune suppression [178].

MSCs can produce a wide range of cells that engage in paracrine signalling, including IL-6 and IL-8, advancing the development of cancer and enhancing chemoresistance [179]. When exposed to cisplatin, instead of going through apoptosis, a subpopulation of cisplatin-resistant MSCs activate a phenotype linked to senescence [179]. As a result, various proteins (such as PLC-y1, RSK1/2/3, WNK1, c-Jun and p53) become phosphorylated, activating signalling pathways resulting in the secretion of IL-6 and IL-8 into the TME. When breast cancer cells and MSCs were simultaneously co-cultured, the therapeutic impact was diminished in vivo due to the upregulation of resistance-related genes (such as MUC1, MYC and BRCA1) in the breast cancer cells after cisplatin pre-treatment [179]. 

MSCs can differentiate into CAFs. Recently, it was revealed that CAF triggered TKI resistance in HCC via the activation of PI3K/Akt/mTOR and RAF/ERK/STAT3 pathways [180]. Moreover, it was determined that the major signalling pathway activated by CAF is STAT3, driving everolimus resistance in neuroendocrine tumours cells [181]. 

In oesophageal squamous cell carcinoma, PAI-1 secreted by CAF activate the MAPK and Akt pathways in a paracrine manner resulting in the production of ROS and the induction of DNA damage and cell death leading to chemoresistance [182]. Additionally, drug resistance was promoted in tumour cells via NF-κB pathway induction by CAF-derived paracrine signals, such as exosomes, metabolites and chemoattractant cytokines [183,184]. CAFs can also enhance stemness through NF-κB signalling activation in gastric cancer [185]. Moreover, CAF enhanced the stemness of HCC by activating the Notch1 signalling pathway [186]. Furthermore, recently, it was discovered that INF-γ/STAT1/Notch3 act as a molecular connection between CSCs and CAF using a bioinformatics strategy in TNBC cell lines resistant to doxorubicin [187].

The cellular composition and functional state of the TME will differ depending on the organ in which the tumour is located, as well as on the cancer type and stage, which will affect the delivery of treatment leading to a heterogeneous exposure to anticancer drugs [13,188,189]. TME can be divided into six different types of specialised microenvironments: the hypoxic, immunological, innervated, metabolic, mechanical and acidity niches. All these niches interact together and facilitate the progression and drug resistance of cancer [3].

Depending on their location within the cancer tissue, the cells in the tumour mass grow in a 3D tissue structure and are unevenly exposed to oxygen. As opposed to the tumour core, which is poorly vascularized, blood vessels in tumour tissues are typically randomly arranged and only cover the outer portion of the tumour mass [189]. A hypoxic microenvironment is created within the tumour core as a result of increased tumour cell proliferation, which places the cells there further away from the supporting blood vessels than the cells outside the tumour. This can result in varied treatment responses. By increasing the expression of genes linked to cell survival, angiogenesis and anti-apoptotic pathways, tumour cells respond to hypoxic circumstances and modified TME leading to the progression of cancer and the development of treatment resistance [190,191,192].

Interestingly, cancer cells may proliferate and colonise in anatomical areas that are sanctuary sites where medications systemically administered are unable to reach the therapeutic window. The brain’s blood-brain barrier (BBB) and the central nervous system (CNS) are the two most typical examples [193]. Additionally, the peritoneum is another sanctuary site in severe paediatric leukaemia that may be treated with intra-peritoneal chemotherapy and tests that result in the management of preventative emission. Among these sanctuaries, the CNS is conceivably the most resentful therapeutic necessity. The extent of CNS tropism is higher in some types of diseases, including melanoma, lung, breast and kidney cancers. Those sanctuaries are physical barriers that lead to devastating clinical outcomes [194].

The TME causes chemotherapeutic resistance via intrinsic or acquired mechanisms. Cancer dormancy, stemness and progression, as well as intercellular communication, redox adaptability and drug resistance, are reprogrammed by hypoxia [195]. Hypoxia affects the TME and treatment efficacy by encouraging cancer cells’ greater production of hypoxia-inducing factors (HIFs), most frequently HIF-1α. This latter stimulates the transcription of numerous genes, including vascular endothelial growth factor (VEGF), which enhance angiogenesis and, as a result, cancer cells are better able to sustain their oxygen supply and metabolism, improving their chances of surviving [196,197]. Increased somatic mutational burden of oncogenes and tumour suppressors, such as TP53, MYC and PTEN, is also linked to the hypoxic niche [198]. Cancer cells with p53 mutations or suppressed p53 transcription have the ability to avoid p53-mediated apoptosis pathways under hypoxic conditions, leading to the selection of cancer cell clones and the production of apoptosis-resistant cells [199]. Under hypoxic conditions, it has been demonstrated that p53 transcriptional activity is inhibited and the expression of efflux pumps, ABCB1 and ABCB5, is increased once HIF-1α binds to p53 in ovarian cancer cells, promoting their resistance to commonly used chemotherapeutics [200]. 

One of the characteristics of cancer is metabolic reprogramming, which is a modification in metabolism or nutrition supply. Increased metabolism of glutamine, glucose, amino acids, lipids, addiction to ROS and accumulation of lactate are common characteristics of cancer [201,202,203]. The synthesis of brain-derived neurotrophic factor by CAFs was driven by lactate in cancer cells in an NF-κB-dependent way, which in turn activated TrkB/Nrf2 signalling in cancer cells to lessen their susceptibility to anlotinib [204]. These results support the connection between drug resistance, metabolism and NF-κB signalling.

Cancer is characterized by dysregulated pH, which is one of the TME variables. Extracellular pH (pHe 7.3–7.5) is often higher than intracellular pH (pHi 6.8–7.2) in healthy tissues and cells, while cancer cells generate a “reversed pH gradient” with increased internal pH and decreased external pH [205,206,207]. This reversed pH gradient makes it difficult for cancer cells to undergo apoptosis and prevents them from dying off [208,209]. Cancer cells’ acidic extracellular environment (pH 6.5–7.1) plays a role in their chemotherapy resistance [210]. Recent research showed that an acidic tumour environment promotes cellular stemness and increases radio- and chemoresistance in oral cancer cells by causing increased cancer cell migration [211]. Acidic environments are extremely stressful for cells triggering many signalling pathways, likely activating powerful survival signalling pathways, such as those linked to cell stemness and undifferentiation leading to an increase in treatment resistance. Melanoma, neuroblastoma and breast cancer cells become more invasive and undergo an increase in oxidative phosphorylation and EMT in an acidic niche [212,213,214]. The development of acidic niches is also influenced by the activation of oncogenes, such as Ras and Myc, and the inactivation of tumour suppressors such as p53. Acidic pHe produces resistance to daunorubicin by inducing the activation of P-gp and the subsequent activation of p38 MAPK [215,216]. Inhibition of apoptosis in colon cancer cells is also associated with tumour acidity and p53 function loss [217]. Moreover, the absorption and resistance to cisplatin in melanoma cells are influenced by an acidified TME [218].

Neurology and cancer sciences are closely related, with neurotransmitters and neuropeptides generated from the nerve creating an “innervated niche” [219,220]. The neuroligin-3 (NLGN3)-stimulated PI3K/mTOR pathway, which is activated by active neurons, aids in the formation of high-grade gliomas [221]. Paracrine stimulations of cGAMP to astrocytes, cytokines production, the activation of the STING pathway and NF-κB and STAT1 signalling are triggered in brain metastatic cells via gap junctions between astrocytes and lung/breast cancer, which promotes cancer growth and resistance to chemotherapy [222,223].

The creation of a mechanical niche depends on stromal cells, extracellular and intracellular components and intercellular signalling [224]. There are various structural proteins in the ECM such as collagen, laminins, fibronectin, elastin, proteoglycans and glycoproteins. The ECM is a 3D network of macromolecules that provides the biochemical and biophysical characteristics of the non-cellular bulk surrounding the cells. Additionally, non-malignant tumour-associated stroma cells are a crucial component of the TME, altering tumour characteristics, illness prognosis and therapeutic response. Cell surface proteoglycans, cell adhesion molecules such as integrins, and hyaluronic acid receptors such as CD44, mediate biochemical and biophysical signalling as well as cell anchoring to the ECM [189,225]. For instance, in breast cancer increased laminin-mediated signalling and overexpression have been connected to diminished treatment responsiveness and improved tumour cell invasion and metastasis [226]. Fibronectin-integrin β1 interactions activate the PI3K/Akt and MAPK/ERK 1/2 pathways leading to chemotherapy resistance [227]. The integrin β1 downstream kinases FAK and Src are activated in HER2+ breast cancer cells that are resistant to lapatinib (a HER2-targeted therapy), resulting in these overcoming HER2 inhibition [228].

Matrix cells in the TME communicate with cancer cells through exosomes. Exosomes are small, bilayered molecules involved in autocrine, paracrine and endocrine signalling that are released by stromal and cancer cells in the TME. Altering vital survival signal transduction pathways, inducing EMT, activating anti-apoptotic pathways and modifying the immune system are just a few of the ways that exosomes can make tumour cells resistant to treatment [229]. The exosome-mediated transfer of different ncRNAs, such as lncRNAs and miRNAs, may be a way for cancer cells to develop treatment resistance by causing genetic and epigenetic changes [229,230]. Recently, it was shown that miR-1228-3p carried by CAF-derived extracellular vesicles increases HCC’s chemoresistance by activating the PI3K/Akt pathway [231]. It was also revealed that Wnt/β-catenin and BMP signalling diminish the susceptibility of hepatoma cells to sorafenib and promote EMT in CAFs-derived Gremlin-1-rich exosomes [232]. Moreover, it was demonstrated that CAF-derived exosomes harbouring miR-20a can encourage chemoresistance and aggressive growth in NSCLC cells via the PTEN/PI3K/Akt signalling pathway [233]. Exosomal miR-21 and IL-6 produced from CAFs together increased MDSC formation in oesophageal squamous cell carcinoma by activating STAT3, which made tumour cells resistant to cisplatin [234]. Furthermore, SOX2 and PD-L1 expression was mediated by PI3K/Akt signalling pathway activation and was shown to be a mechanism by which exosomes from CRC/MDR cells may increase cetuximab resistance in KRAS wild-type cells [235].

### 3.11. Epithelial-Mesenchymal Transition

The phenotypic change from epithelial to mesenchymal cells, or epithelial-mesenchymal transition (EMT), occurs when epithelial cells lose their cell identity and take on mesenchymal traits, altering the cell’s shape and expression of surface markers in the process [236]. Epithelial cells, in the EMT process, experience depolarization, lose their cell-cell contact and adherent property and develop elongated fibroblast-like morphology, which is known to be triggered by ncRNAs, growth factors, cytokines and hypoxia. These occurrences are accompanied by a concurrent increase in mesenchymal markers (integrin, laminin 5, N-cadherin, fibronectin, vimentin and type I collagen) and a concurrent decrease in epithelial markers (laminin 1, desmoplakin, E-cadherin and type IV collagen) expression. EMT is typically seen under healthy conditions, but tumour cells have the ability to carry out the same process while cancer is developing. Recent evidence suggests that pathological hyperactivated EMT is closely linked to elevated therapeutic resistance in cancer cells. Intracellular regulatory miRNA, exogenous inducers, epigenetic modulators and cellular signalling pathways such as SMADs, PI3K, MAPK, ERK, TGF-β, Notch and Wnt/β-catenin are only a few of the molecular players involved in the regulation of EMT [237,238]. For instance, through the Wnt/β-catenin pathway, tongue squamous cell carcinoma cells gained cisplatin resistance and stem cell-like properties, resulting in an enhanced EMT [239]. Moreover, in oral cancer, Notch signalling increases the population of CSCs, improves angiogenesis and EMT, and strongly responds to the DNA damage response induced by cisplatin [240]. TGF-β is the primary substance released by CAFs; it causes EMT and encourages the acquisition of gastric CSC features, both of which eventually result in drug resistance [241]. Furthermore, miR-155 is overexpressed in oral squamous cell carcinoma, which results in resistance to cisplatin by inhibiting the expression of FOXO3a and promoting the EMT pathway [242].

Recently, it has been demonstrated that cancer cells treated with chemotherapy release IL-1β, triggering the release of integrin-αvβ1 and matrix metalloproteinase 9, causing the activation of TGF-β, which in turn promotes EMT in breast cancer cells [243]. Moreover, the family with sequence similarity 46, member A (FAM46A), activated TGF-β pathways, promoting chemoresistance in ovarian cancer cells [244]. TGF-β signalling promoted EMT and resistance to doxorubicin in breast cancer cells by upregulating lncRNA urothelial carcinoma-associated 1(lncRNA UCA1) [245]. HIF-1α/TGF-β2/GLI2 signalling is responsible for chemoresistance in CRC cells [246].

It was shown that hexokinase domain containing protein-1 (HKDC1) is essential for gastric cancer cell glycolysis, carcinogenesis and EMT by activating the NF-κB pathway, resulting in resistance to 5-FU, oxaliplatin and cisplatin in gastric cancer patients [247]. For instance, epithelial ovarian cancer (EOC) cells resistant to paclitaxel, cisplatin, erlotinib and carboplatin displayed high NF-κB activity [248]. The Notch signalling pathway is upregulated in breast cancer patients that are resistant to tamoxifen, which can promote CSCs and EMT [249]. Furthermore, as a result of the activation of PI3K/Akt/mTOR signalling, the expression of EMT and CSC markers was considerably increased in cisplatin-resistant EOC cells [250].

### 3.12. Multidrug Resistance

MDR is a common problem in cancer patients undergoing long-term chemotherapy and is the primary cause of death. Some tumours that become resistant to one type of drug are also found to be resistant to different drugs, despite the fact they might have different modes of action from the primary therapy. In fact, cross-resistance to a variety of anticancer medications with unique structural and functional characteristics is a hallmark of the MDR phenotype. As discussed in the sections above in more detail, host factors, tumour factors and tumour-host interactions are just a few of the many variables influencing drug resistance, but also MDR. 

Genetic variations and drug-drug interactions are examples of the hosts’ contributing elements. Genetic variants, such as single nucleotide polymorphisms (SNPs), copy number variations, insertions, deletions and repeats in genes encoding drug targets, DNA repair, cell cycle control, drug efflux and enzymes that are related to drug metabolism, can affect drug efficacy [12]. Drug-drug interactions can change drug efficacy by interfering with the drug’s pharmaco-kinetics and -dynamics when the cancer patient, at the same time of their treatment, is administrated another drug, herbal supplement or is exposed to an environmental factor (e.g., diet, smocking, exposure to chemicals) [251].

Examples of tumour-related MDR factors are impaired influx transporters that promote a reduction in drug uptake, while overexpression of MDR efflux transporters of the ABC superfamily can trigger an increase in drug efflux [12,252,253]. Other examples of tumour MDR factors include exocytosis of drug-loaded lysosomes and extracellular vesicles, which mediate drug efflux, improved DNA damage repair, deregulation of anti-apoptotic cell death processes, deregulation of miRNA and/or epigenetic changes, as well as the adaptability of CSCs and intratumour dynamics and heterogeneity [12]. 

Selective pressure from various agents and stressors, TME, acidic pH and the intracellular transmission of characteristics carried out by extracellular vesicles are a few examples of the interactions between the tumour and the host that can promote MDR [12].

## 4. Altered Signalling Pathways Involved in Drug Resistance to Cancer

Cellular signalling is an intracellular network of related crosstalking molecules ensuring cellular homeostasis. A potent stimulus activates a molecular receptor promoting a downstream signalling cascade that will determine the cellular function. As discussed above, genetic and epigenetic modifications of certain molecular components of these signalling pathways (e.g., RTK) can lead to their dysregulation, which causes cancer progression and drug resistance.

In cancer, oncogenic pathways are abnormally active whilst tumour suppressor pathways are inhibited. The abnormal activation or inhibition of one or more signalling pathways can have a pivotal role in cancer drug resistance. Below, we discuss some of the key downstream pathways involved in drug resistance in response to altered upstream receptors.

### 4.1. Wnt/β-Catenin Pathway

It has been discovered that EMT and resistance to chemotherapeutic drugs in cancer cells depend on the zinc-finger transcription factor pleomorphic adenoma gene like-2 (PLAGL2). Recently, it has been demonstrated that through the activation of the Wnt/β-catenin signalling pathway, PLAGL2 encourages adriamycin resistance and the aggressiveness of cells in breast cancer [254]. A recent study on squamous transitioned lung cancer suggested that Wnt signalling may have a role in increasing adeno-to-squamous transdifferentiation (AST) [255]. For instance, the upregulation of the Wnt pathway was detected in transitioned lung cancer following osimertinib resistance. Various investigations have shown the significant role that AST has played in the development of resistance to molecular-targeted therapy in lung cancer. Recently, it has been found that the ROS-Wnt axis acts as the AST tipping point and plays a crucial role in dynamically managing the homeostasis between the adeno- and squamous-specific transcription factors networks [256]. Wnt signalling is the primary regulator of the CSC gene expression program. Wnt3a has been reported to be able to activate p38 MAPK. The latter has been demonstrated to interact with the Wnt/β-catenin pathway and functions as a β-catenin chromatin-related kinase, which is essential for controlling the signalling system involved in tumour growth, metastasis and chemoresistance in CRCs [257]. Moreover, recent research suggests that SNORD1C (small nucleolar RNAs) promotes cancer cell stemness and drug resistance in CRC via the Wnt/β-catenin pathway and may serve as a biomarker that predicts the prognosis and aggressiveness of this cancer [258]. It was demonstrated how IL-6/STAT3 signalling is activated by Hsp90 (heat shock protein) inhibitor therapy through the actions of ERK and Akt, which subsequently activate the Wnt signalling pathway, allowing NSCLC cells to develop CSC characteristics and resistance to Hsp90 inhibitor [259].

### 4.2. The JAK/STAT Signalling Pathway

It was discovered that miR-106a-3p is an oncomiR in gastric cancer that triggers apatinib resistance due to the overexpression of JAK2/STAT3 proteins and their signalling [260]. Moreover, through the PTEN/Akt/SMAD2 and RAS/MEK/FOS MAPK/Akt pathways, the apurinic/apyrimidinic endodeoxyribonuclease 1 (APEX1)/miR-27a-5p axis contributed to the resistance to doxorubicin in gastric cancer cells [261]. Recently, it was discovered that chronic myeloid leukaemia (CML) cells with a high amount of intracellular angiopoietin-1 (iANG-1) were resistant to dasatinib, nilotinib, imatinib and other TKIs. Furthermore, a unique drug-resistant mechanism in CML was revealed by the substantial upregulation of the p-SRC/p-STAT5 axis by iANG-1 [262].

### 4.3. PI3K/Akt/mTOR Pathway

Proprotein convertase subtilisin/kexin type 9 (PCSK9), a crucial enzyme for antitumour immune responses, also activated Akt by suppressing PTEN, which caused HCC to develop sorafenib resistance [263]. Furthermore, nuclear paraspeckle assembly transcript 1 (NEAT1), which is a lncRNA, activated the c-MET/Akt pathway via miR-335 in HCC cells, resulting in sorafenib resistance [264]. Recently, it was elucidated that in male HCC and hepatoma cell lines, the NEAT1v1-superoxide dismutase 2 (SOD2) axis confers lenvatinib and sorafenib resistance and shifts the growth mode from MEK/ERK-dependent to Akt-dependent mode [265]. Lenvatinib’s lethal effects were amplified by the MEK inhibitor selumetinib, which implies that thyroid cancer cells convert from Akt-dependent to MEK/ERK-dependent cell growth modes to develop resistance to Lenvatinib [266]. Acid-sensing ion channel 1a (ASIC1a) is an H+-gated cation channel that promotes tumour cell invasion and migration. ASIC1a is highly expressed in resistant HCC cells. ASIC1a-induced calcium influx activates the PI3K/Akt pathway, leading to drug resistance in resistant HCC cells [267].

### 4.4. MAPK Pathway

Recently, it was demonstrated that mitochondrial fusion dramatically decreases the susceptibility of breast cancer cells to tamoxifen under metabolic stress and likely contributes to the development of acquired drug resistance through AMPK and MAPK signalling [268]. Through p44/42 MAPK-Drp1 (a dynamin-related GTPase) signalling, membrane-bounded G-protein coupled oestrogen receptor (GPER) causes mitochondria fission, which is essential for GPER-induced cell apoptosis in breast cancer cells [269]. According to pertinent studies, one of the key mechanisms by which CRC cells develop resistance to cetuximab is the activation of the RAS/RAF/MEK/MAPK pathway [270]. Fucosyltransferase VI (FUT6) modulates the EGFR/ERK/STAT signalling pathway to control head and neck squamous cell carcinoma invasion, migration, proliferation and EGF-induced EMT [271]. Moreover, it was recently identified that resistance to gefitinib and osimertinib in NSCLC cells is driven through the cholesterol/EGFR/Src/ERK/SP1 axis [272].

## 5. Strategies to Overcome Drug Resistance in Cancer

**Figure 3:**
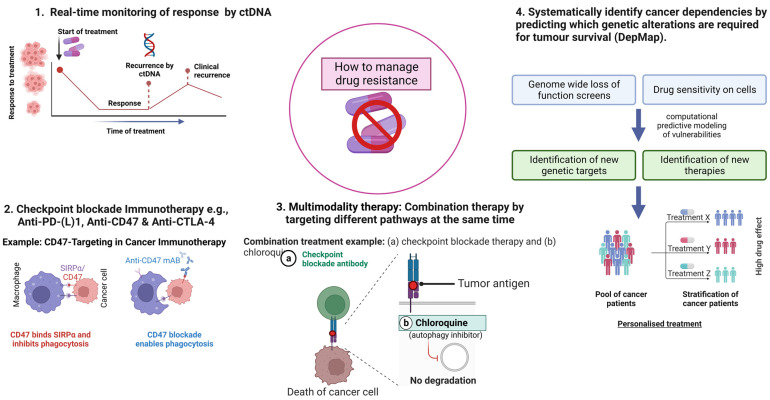
**Drug resistance management.** Strategies used to manage and overcome drug resistance are depicted in this picture. (1) Earlier detection of actionable genomic modifications using ctDNA is a powerful tool to predict cancer recurrence influencing more effective treatment decision-making that results in a better response to treatment. (2 and 3) Immunotherapy, such as checkpoint inhibitors, can be used as monotherapy or in combination to simultaneously target multiple pathways and increase treatment effectiveness. (4) Mapping cancer dependencies using DeepMap is an effective approach to predicting genes responsible for drug resistance and identifying new genetic targets, thereby facilitating the discovery of drugs that can potentially overcome resistance. Created with BioRender.com.

It will be very challenging to choose the optimal approach to combat drug resistance due to the high complexity and heterogeneity of tumours. Here, we state strategies used to manage drug resistance and present how the deployment of cutting-edge diagnostic and therapeutic technologies are used to prevent its emergence (Figure 3).

### 5.1. Circulating Tumour DNA

Liquid biopsy has received a lot of interest in oncology diagnosis over the past several years. It is a biological sample approach that offers details on the real-time dynamics of tumour biomarkers in a quick, cheap, easy to access, minimally invasive and patient-friendly way. Several soluble components associated with tumour genetics include exosomes, circulating tumour cells, cell-free DNA (cfDNA) and circulating tumour DNA (ctDNA). In the latter, genetic modifications associated with cancer can be detected, such as amplification, point mutations, aneuploidy, rearrangements and patterns of fusion and methylation. By using platforms based on the polymerase chain reaction (PCR) and next-generation sequencing (NGS), liquid biopsy can be analysed to depict the current complexity of the patient’s overall tumour mass [273,274]. ctDNA amount may serve as a prognostic indicator as its analysis may reveal the factors affecting prognosis. Following surgical resection, ctDNA is sensitive enough to detect minimal residual disease (MRD). Following surgery, ctDNA analysis offers a good prognostic evaluation and can help identify patients who have a very high risk of recurrence, potentially avoiding unnecessary chemotherapy. Moreover, according to genetic changes, tailored treatments can be created using ctDNA. Patients whose BRAF V600E was missed in tissue analysis due to spatial heterogeneity can have their BRAF V600E found in their plasma using ctDNA. Therefore, offering the opportunity for BRAF inhibitors administration in combination with anti-EGFR monoclonal antibodies, for example, in CRC patients with a BRAF V600E mutation [273]. This offers evidence that ctDNA screening is useful and equivalent to tissue-based biomarker screening for choosing treatments. Therefore, it may be possible to use ctDNA as a surveillance tool to spot clones that are developing resistance to current treatments and provide a chance to convert therapy early on before the disease progresses.

### 5.2. Immunotherapy

Moreover, immunotherapy, which includes cancer vaccines, monoclonal antibodies and inhibitors of immune checkpoints such as anti-PD-1/PD-L1 and anti-cytotoxic T-lymphocyte-associated protein 4 (CTLA4) [275,276], is promising in that it may revolutionise the treatment of cancer by inducing, enhancing or suppressing immune responses against cancer cells. Recently, anti-CD47 agents have gained attention. Numerous tumour cell surface membranes have high levels of CD47 expression, which controls macrophage phagocytosis by binding SIRPα to prevent the eradication of host cells. Inhibiting the interaction between cancer cells and macrophages and inducing phagocytosis may be achieved by CD47-blocking drugs, such as monoclonal antibodies that target CD47/SIRPα [277]. Thus, combining immunotherapy and chemotherapy can be an effective approach to overcoming drug resistance. For example, recent clinical trial results for unresectable HCC have shown that combination therapies, such as tremelimumab (anti-CTLA4 Ab) (HIMALAYA) + durvalumab (anti-PD-L1 Ab) + bevacizumab (anti-VEGF Ab) (IMbrave 150) + atezolizumab (anti-PD-L1 Ab) outperform monotherapy in terms of clinical outcomes [278,279]. Interestingly, it was recently shown that DTPs cells and EGFR TKI-resistant cells are effectively eliminated by CD70-targeting chimeric antigen receptor (CAR) T and NK cells and anti-CD70 antibody drug conjugates. These findings point to CD70, a cell surface protein, as a promising therapeutic target for EGFR mutant tumours that have developed acquired resistance to EGFR TKI [280]. Moreover, Akt inhibition can specifically result in a favourable immunological profile in the TME of the breast, including an enhanced density of CD3+CD8+ cells and improved IFN genes expression, offering a justification for utilising Akt inhibition and immunotherapy in combination [281]. In PTEN-deficient xenografts, AZD8186 (a PI3Kβ inhibitor) improved anticancer activity in combination with anti-PD-1 Ab [282].

Furthermore, in cancer treatment, the combination of cytotoxic medicines and autophagy inhibitors such as chloroquine (CQ) and its derivative is gaining more attention. Recently, it was demonstrated that CQ promotes colon cancer cells to become more sensitive to 5-FU via inhibiting ataxia telangiectasia and Rad3-related (ATR) kinase-mediated HIF-1α translation and interfering with HIF-1α’s hypoxic function [283]. Moreover, recent data implied that the inhibition of autophagy with CQ could circumvent in TNBC, a therapeutic resistance mechanism to PI3K/Akt inhibitors with paclitaxel, making the assessment of such combinations in clinical trials justified [284]. Dong et al., in 2023, presented a drug loading system that combines CQ and a CD47 antibody (aCD47) with a bionic lipoprotein (BLP) carrier (BLP-CQ-aCD47) to improve drug delivery, cancer immunotherapy and potentially help to overcome drug resistance [285].

### 5.3. Nanotechnology

Nanoparticle-based medications have effectively decreased side effects, eliminated drug resistance and increased medicinal efficacy [286]. The selectivity of the target gives nano-based medications an edge over traditional therapy [287]. Numerous nanoparticles, including mesoporous silica, metal and polymeric nanoparticles, as well as micelles, liposomes, dendrimers and nanostructured lipid carriers, have been created and investigated over time and have significantly reduced chemoresistance in cancer [288]. The newly created doxorubicin-melittin polymersome (Dox-Mel PL) drug delivery system was capable of controlling MDR cancer cells and offered the following benefits: (1) biocompatible polymersome (a poly lactic acid-hyaluronic acid (20k–10k) di-block copolymer) promote synergistic effects of the simultaneous administration of Dox (anticancer agent) and Mel (a major component of bee venom); (2) reduction of Dox and Mel side effects; and (3) downregulation of P-gp by Mel prevent drug resistance. Dox-Mel PL overcomes MDR through P-gp inhibition and PI3K/Akt/NF-κB pathway downregulation [289]. Moreover, by inhibiting the NF-κB expression and activation and downregulating PD-L1 level, Ab-G/S-NP (nanoparticles that are loaded with sorafenib and GSK1059615) controlled the activation of cellular signalling pathways in HCC-resistant cells to overcome their drug resistance to sorafenib. For the purpose of creating a more potent treatment for sorafenib-resistant malignancies, these findings call for additional research on the combination of treating HCC-resistant cells with GSK1059615 (a PI3K/mTOR inhibitor) in vivo [290]. Furthermore, an innovative multifunctional medication delivery system based on targeted gold nanoparticles has been created as a useful approach for highly focused and EGFR-TKI-resistant reversal therapy. The nanoparticle (cRGD-GIPG) ensures that the treatment is successfully delivered to the EGFR-TKI-resistant NSCLC by inhibiting the activation of the TGF-β/PDLIM5/SMAD resistance pathway and triggering drug-resistant cells to die by mitochondrial apoptosis. Thus, cRGD-GIPG exhibits strong anticancer effects against EGFR-TKI-resistant NSCLC cells both in vitro and in vivo [291]. Extensive work has also been conducted into finding novel compounds (such as allosteric modulators) [292], creating new biotechnology (such as PROTAC) [293,294,295] and suggesting effective drug combinations that successfully combat drug resistance.

### 5.4. Gene Editing 

It is common practise to utilise high-throughput forward genetic screening methods to investigate the molecular processes behind particular cellular phenotypes, such as treatment resistance in malignancies. To undertake loss-of-function screening across a variety of signalling pathways and biological processes, CRISPR-associated nuclease Cas9 (CRISPR/Cas9) is a particularly successful method. Single or double knockouts or the modification of genes responsible for drug resistance can now be produced using the genome-wide CRISPR/Cas9 gene editing method [296,297]. For instance, lung cancer cell proliferation and EMT were suppressed by PNO1 (RNA-binding protein)/CRISPR/Cas9 through inhibiting the Notch signalling pathway in lung adenocarcinoma. Using CRISPR/Cas9 technology may be a beneficial technique [298].

### 5.5. Computational Strategies 

Moreover, the emergence of deep learning, the vast amount of digital data and powerful computing resources can offer an effective pipeline for enhanced drug discovery, help us understand how drugs become resistant to them and help us make the best decisions possible when treating patients with EGFR-mutated NSCLC [299,300]. Furthermore, Fröhlich et al. recently discussed the second-generation MAPK Adaptive Resistance Model (MARM2.0), which aims to explain how drug-adapted BRAFV600E melanoma cells rewire EGFR/MAPK signalling. MARM2.0 is developed using rule-based modelling in PySB (python program) with thermodynamic balance and builds on an extensive body of theoretical, biochemical and structural work on EFGR/MAPK signalling and feedback regulation [301]. Furthermore, a unique phosphoproteomic-based machine learning technology called VESPA (Virtual Enrichment-based Signalling Protein-activity Analysis) is used to analyse enzyme-substrate connections and measure the activity of signalling proteins. Scientists have used it to investigate the mechanisms of post-translational cell adaptation that cause CRC to be resistant or insensitive to clinically useful targeted therapies [302]. Interestingly, ‘DRESIS’, a comprehensive database describing information on drug resistance, was recently created and is anticipated to have significant effects on clinical treatment optimisation and the discovery of novel drugs in the future [303].

### 5.6. miRNAs

miRNAs can also control drug sensitivity and modulate resistance by post transcriptional gene regulation. Therefore, they do not only serve as biomarkers, but also as drug targets for overcoming drug resistance. For instance, exosomal miR-107 modulated the HMGA2/mTOR/P-gp axis, drastically increasing the susceptibility of resistant gastric cancer cells to cisplatin, indicating that exosomal miR-107 may be a promising target in the therapy of gastric cancer [304]. Recently, the tumour suppressor miR-4486 was used to increase the chemo-sensitivity of cisplatin in gastric cancer. The JAK3/STAT3 pathway was the target of this activity [305]. Moreover, by inhibiting CD44-induced CSC-like features via EGFR-mediated MAPK and Akt signalling pathways, miR-302a has also been discovered to restore the response to cetuximab [306].

### 5.7. Targeting Signalling Pathways

**Figure 4:**
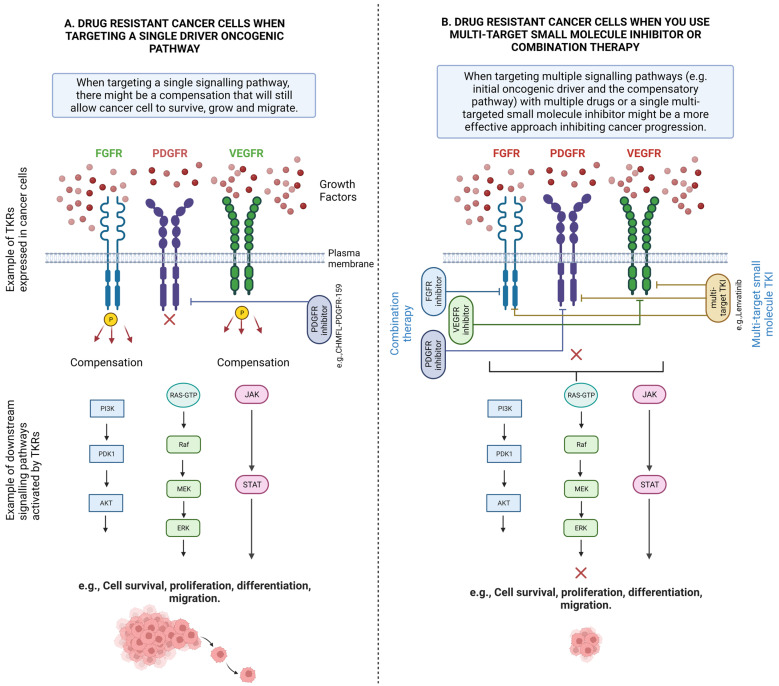
**Targeting multiple pathways in drug-resistant cells.** The impact of many therapies on signalling in drug-resistant cells is depicted in this picture. (**A**) When targeting a single signalling pathway, such as PGFR signalling, cancer cells become resistant to PDGFR inhibitors (such as CHMFL-PDGFR-159) by activating compensatory signalling pathways of alternative RTKs (e.g., FGFR or VEGFR) leading to cell survival and migration. (**B**) However, resistance and signalling reactivation can be overcome by combination therapy and multi-target kinase inhibitors that target multiple signalling pathways leading to effective inhibition of cancer progression and drug resistance. Created with BioRender.com.

PTK overexpression, including HER2, EGFR, FGFR, PDGFR, VEGFR and IGFR, activates numerous cell signalling pathways, including STAT3, NF-κB, PI3K/Akt and ERK1/2. It also results in an aberrant expression of proteins associated with apoptosis in cancer cells, which is a major contributor to chemotherapy resistance in tumour cells. Target therapy directed at specific tyrosine kinases will therefore overcome this resistance. There are various instances where platelet-derived growth factor (PDGF) ligands and receptors are both expressed in malignant cells; nevertheless, PDGF expression and function typically involve the tumour stroma. The pursuit of PDGFR inhibitors represents a successful strategy given the significance of the TME and the critical part that PDGF signalling plays in creating and maintaining that milieu [307]. A reversible and ATP-competitive PDGFR inhibitor called CP-673451 inhibits both PDGFRα and PDGFRβ kinase and effectively suppresses the downstream phosphorylation of PI3K/Akt [308]. Although CP-673451 reduces PDGFR-β expression and tumour growth in Lewis lung carcinoma-carrying mice, it did not increase overall survival when compared to radiation and Endostar combined therapy [309]. In a previous study it was shown that in soft tissue cancers, PDGFRα loss and bypass of the Akt-signalling pathway are one cause of acquired resistance to PDGFR inhibitor via activation of alternative compensatory signalling pathways. This study also outlines that by blocking FGFR1 in vitro this resistance was eliminated. Therefore, as an alternative strategy, combination therapy that simultaneously targets several compensatory signalling pathways can be developed to overcome resistance [310] (Figure 4).

It is interesting to note that, in some cases, resistance to target therapy may develop over time and may be brought on by several translocation break points, various mutations at the target site or abnormalities in the phosphorylation of protein substrates. Therefore, discovering novel combination therapies might reduce drug resistance development. For instance, the inhibition of both PI3K/Akt/mTOR and MAPK pathways presents a viable approach to treat the majority of CRC patients and circumvent potential resistance mechanisms that result from single-target treatment, as revealed for the MEK inhibitor pathway [311]. Moreover, MEK1/2 inhibitor (BAY 86-9766) therapy of multi-drug-resistant human CRC cell lines showed a considerable effect. Cetuximab (EGFR inhibitor)-resistant CRC cells also experienced synergistic apoptotic and antiproliferative effects from combination therapy with BAY 86-9766 and cetuximab through the inhibition of the Akt and MAPK pathways [270]. Furthermore, combining BEZ235 (a dual PI3K/mTOR inhibitor) and cisplatin treatment dramatically reduced the capacity of chemoresistant EOC cells to form colonies, increased ROS levels and increased apoptosis when compared to cisplatin treatment alone. In addition, compared to cisplatin mono-treatment, the combination method efficiently inhibited the PI3K/Akt/mTOR signalling pathway, reversed EMT and lowered the expression of the CSC marker and re-sensitized chemoresistant EOC cells to cisplatin [250]. It was found that imatinib (a TKI) sensitivity is increased, blastic phase of chronic myeloid leukaemia (CML-BP) cells resistant to TKIs are eliminated and leukaemia engraftment is decreased when the integrated stress response (ISR) signalling is inhibited by the small molecule ISRIB in combination with imatinib. It was demonstrated how this dual therapy precisely alters the profile of gene expression and inhibits oncogenic JAK/STAT5 and RAS/RAF/MAPK/ERK signalling. To combat TKI-resistant leukaemic cells that demonstrate RAS/RAF/MAPK and STAT5 signalling hyperactivation due to driver mutations such as PTPN11 (SHP2) that can be detected by NGS analysis, the combination of ISRIB and imatinib as a potential treatment approach was suggested [312].

Inhibiting several signalling targets with a single drug is an alternate strategy for overcoming resistance. For instance, sorafenib is a multitarget kinase inhibitor that targets several RTKs in the cell membranes, such as fibroblast growth factor receptor 1 (FGFR1), PDGFR, VEGFR 1, 2, and 3, FMS-related tyrosine kinase 3 receptor (FLT3), stem cell factor receptor (KIT) and RET proto-oncogene (RET), as well as downstream intracellular serine/threonine kinases, such as B-Raf and Raf-1, and kinases in the Ras/Raf/MEK/ERK signalling pathway. By inhibiting these kinases and the downstream signalling molecules in a variety of oncogenic pathways, tumour cell apoptosis, proliferation and angiogenesis are all markedly reduced [313,314,315,316]. However, within 5 years of surgery, 70% of HCC patients who received adjuvant sorafenib treatment following surgical resection or local ablation (or both) experienced tumour recurrence, and the majority of these recurrent HCCs were sorafenib-resistant [317]. There is growing evidence that acquired sorafenib resistance is significantly influenced by the IGF/FGF axis, an upstream Akt regulator, such as in HCC [318]. It was discovered that ceritinib, an IGFR inhibitor first used to treat NSCLC, could sensitise HCC cells to sorafenib both in vitro and in xenograft and HCC mice models by inhibiting the IGF-1R/Akt pathway [319]. Compared to when it is taken alone, ceritinib significantly inhibits the proliferation of HCC cells when combined with sorafenib. Additionally, it was discovered that the linsitinib (IGFR inhibitor) and the brigatinib (FGFR inhibitor) were successful in reducing the viability of sorafenib-resistant HCC cells via the Akt pathway [318]. Recently, a study showed that niclosamide, through modulation of IGF-1R/p-IGF-1R/stemness and metabolic alterations, can boost sorafenib sensitivity in sorafenib-resistant HCC cells/organoids. For sorafenib-resistant HCC cells, combining sorafenib and niclosamide can result in a synergistic combination index that lowers IGF-1R/p-IGF-1R/OCT4 expression. Niclosamide significantly increased the capacity of sorafenib to decrease the mitochondrial membrane potential in vitro by substantially downregulating the sorafenib-induced gene expression of stemness (OCT4), drug resistance (ABCG2) and glycolysis (GLUT1, HK2, LDHA and PEPCK) [320]. Growing data suggest that niclosamide, an antihelminthic drug, has the potential to be a novel treatment for diseases such as cancer other than helminthic disease, since it is a multifunctional medication that may interfere with a variety of biological processes and signalling pathways [321]. The growth of tumours in several cancers, including drug-resistant HCC, prostate and oesophageal cancer, has been shown to be inhibited by niclosamide [322,323,324]. Moreover, lenvatinib and MEK inhibitors were evaluated in vitro and in vivo for the treatment of anaplastic thyroid cancer. Based on decreased tumour proliferation and increased apoptosis, which are caused by the Akt and ERK signal pathways, they discovered that the combination revealed synergistic effects and strengthened the anticancer impact [266]. Furthermore, immense effort has gone into several ongoing clinical trials (Table 1) aiming to evaluate the clinical benefits of various novel combination therapies to overcome drug resistance. 

Despite the notable developments and significant progress already mentioned in this section, there are still restrictions due to the scarcity of biomarker data, which makes designing clinical trials more difficult. These challenges emphasise the need to consider the advancement of artificial intelligence methodologies and their incorporation into clinical practise which can help doctors in patient management. Moreover, designing trials based on aberrant molecular pathways and genomic profiling (Table 1), as well as an understanding of how genetic variations involved in the medication mechanism of action, absorption, metabolism and elimination affect treatment response and the likelihood of serious adverse drug reactions. The impact of real-time tracking on patient health and prognosis will be possible by combining the aforementioned strategies and taking into account the unique characteristics of each tumour.

## 6. Conclusions

Each tumour has a unique collection of traits that govern how it progresses. Despite the challenges, it is still possible to sketch out a strategy for combating the issue of cancer drug resistance by employing our knowledge of its biological constituents and moving to a more personalised approach to treatment. This informative and comprehensive review highlights the importance of understanding the tumour properties, its drivers and dependencies to effectively block one or more signalling pathways to stop cancer progression and prevent resistance to treatment. Real-time monitoring of cancer progression, checkpoint blockade immunotherapy, multimodality therapy and systematically identifying cancer addictions are progressive steps towards effectively eradicating cancer without giving it a chance to adapt and utilise compensatory mechanisms. A combination of drugs targeting multiple pathways along with the utilisation of computational strategies that foresee cancer growth and signalling adaptability will be a promising approach to reduce the likelihood of developing drug resistance in the future.

## Figures and Tables

**Table 1:** Recruiting phase 3 interventional clinical studies targeting receptors tyrosine kinase signalling pathways in drug-resistant cancers. Interventions targeting RTK signalling are highlighted in bold.

Interventions	NCT Number	Type of Cancer	Study Title
Targeting multiple RTKs			
**Famitinib (a RTKI against multiple targets, e.g., VEGFR 2/3, PDGFR and stem cell factor receptor (c-kit)).** **Sunitinib (a RTKI against multiple targets, e.g., VEGFR and PDGFR)**	NCT04409223	Gastrointestinal Stromal Tumours	Efficacy and Safety of Famitinib Versus Sunitinib in the Treatment of Advanced Gastrointestinal Stromal Tumour Patients After Failure of Imatinib
**AL3818 (a RTKI against multiple targets, e.g., VEGFR, FGFR, PDGFR and c-kit).** Paclitaxel, Pegylated Liposomal Doxorubicin (PLD), Topotecan, Carboplatin	NCT02584478	Endometrial Carcinoma, Ovarian Carcinoma, Fallopian Tube Carcinoma, Primary Peritoneal Carcinoma, Cervical Carcinoma	Phase 1/2a/3 Evaluation of Adding AL3818 to Standard Platinum-Based Chemotherapy in Subjects With Recurrent or Metastatic Endometrial, Ovarian, Fallopian, Primary Peritoneal or Cervical Carcinoma (AL3818-US-002)
**Regorafenib (a RTKI against multiple targets, e.g., VEGF1/2/3, PDGFR, FGFR, KIT, RET, RAF-1, BRAF)** Nivolumab, Docetaxel, Paclitaxel, Irinotecan, Trifluridine/Tipracil	NCT04879368	Gastro-Oesophageal Cancer	RegoNivo vs Standard of Care Chemotherapy in AGOC
Targeting EGFR			
**ASK120067 (Third generation EGFR TKI)** **Gefitinib (First generation EGFR TKI)** ASK120067, Placebo Gefitinib 250 mg, Gefitinib, Placebo ASK120067	NCT04143607	Locally Advanced or Metastatic NSCLC	ASK120067 Versus Gefitinib as First-line Treatment for EGFRm Locally Advanced or Metastatic NSCLC
**Aumolertinib (Third generation EGFR TKI)** **Osimertinib (Second generation EGFR TKI)** Pemetrexed, Cisplatin, Carboplatin. Paclitaxel, Nab paclitaxel, Gemcitabine	NCT05493501	NSCLC	Aumolertinib With Chemotherapy or Alone Compared With Osimertinib in Patients With Epidermal Growth Factor Receptor-Mutant Non-Small Cell Lung Cancer
**Gefitinib (First generation EGFR TKI)** **Afatinib (Second generation ErbB family inhibitor** **Erlotinib (First generation EGFR TKI)** Metformin Hydrochloride, Placebo	NCT05445791	NSCLC	Metformin Plus Tyrosine Kinase Inhibitors for Treatment of Patients With Non-small Cell Lung Cancer With EGFR Mutations
Targeting EGFR and HER-2			
**Pyrotinib (EGFR and HER2 inhibitor)** **Trastuzumab (HER2 inhibitor)**	NCT05346861	HER2-positive Breast Cancer, Metastatic Breast Cancer	Pyrotinib Rechallenge in Her2-positive Metastatic Breast Cancer Pretreated With Pyrotinib and Trastuzumab
Targeting EGFR and VEGFR			
**Gefitinib (First generation EGFR TKI)** **Apatinib (VEGFR-2 TKIs)** Placebo	NCT02824458	Non-Squamous NSCLC	A Study of Gefitinib With or Without Apatinib in Patients With Advanced Non-squamous Non-Small-Cell Lung Cancer Harboring EGFR Mutations
**Osimertinib (Second generation EGFR TKI)** **Bevacizumab (anti-VEGF monoclonal antibody)**	NCT04181060	Advanced Lung Non-Squamous Non-Small Cell Carcinoma, Metastatic Lung Non-Squamous Non-Small Cell Carcinoma, Recurrent Lung Non-Squamous Non-Small Cell Carcinoma, Stage IIIB Lung Cancer AJCC v8, Stage IV Lung Cancer AJCC v8	Osimertinib With or Without Bevacizumab as Initial Treatment for Patients With EGFR-Mutant Lung Cancer
Targeting BCR-ABL and JAK			
**Dasatinib (small-molecule, BCR-ABL inhibitor)** **Ruxolitinib (JAK inhibitor)** Prednisone, Vincristine, Daunorubicin. Pegaspargase. Erwinase^®^, Cyclophosphamide, Cytarabine, Mercaptopurine, Methotrexate, Blinatumomab, Bortezomib, Dexamethasone, Doxorubicin, Etoposide, Clofarabine, Vorinostat. Idarubicin. Nelarabine, Thioguanine, Asparaginase Erwinia chrysanthemi (recombinant)-rywn, Calaspargase Pegol	NCT03117751	Acute Lymphoblastic Leukemia, Acute Lymphoblastic Lymphoma	Total Therapy XVII for Newly Diagnosed Patients With Acute Lymphoblastic Leukemia and Lymphoma
Targeting VEGF pathway			
**IBI305** **(anti-VEGF monoclonal antibody)** Sintilimab, Pemetrexed, Cisplatin, Placebo1, Placebo2	NCT03802240	Non-Squamous NSCLC	Sintilimab ± IBI305 Plus Chemotherapy (Pemetrexed + Cisplatin) for EGFRm + Locally Advanced or Metastasis Non-Squamous NSCLC Patients After EGFR-TKI Treatment Failure
**BD0801** **(anti-VEGF monoclonal antibody)** Paclitaxel, Placebo, Topotecan, doxorubicin liposome	NCT04908787	Ovarian Cancer	A Phase III Study of BD0801 Combined With Chemotherapy in Recurrent, Platinum-resistant Epithelial Ovarian Cancer
Targeting mTOR			
**Everolimus (mTOR inhibitor)** Letrozole, Bicalutamide, Itraconazole	NCT03458221	Recurrent Ovarian Cancer, Signal Transduction Pathway Deregulation, Therapy-Associated Cancer	Signal TrAnsduction Pathway Activity Analysis in OVarian cancER
Targeting HER3			
**Patritumab Deruxtecan** **(HER3-DXd antibody attached to topoisomerase I inhibitor)** Platinum-based chemotherapy	NCT05338970	Non-Squamous NSCLC, EGFR L858R, EGFR Exon 19 Deletion	HERTHENA-Lung02: A Study of Patritumab Deruxtecan Versus Platinum-based Chemotherapy in Metastatic or Locally Advanced EGFRm NSCLC After Failure of EGFR TKI Therapy

## Data Availability

Not applicable.

## Abbreviations

ABC	ATP-binding cassette
ABCC5	ATP binding cassette subfamily C member 5
aCD47	CD47 antibody
AhR	Aryl hydrocarbon receptor
ALK	Anaplastic lymphoma kinase
APEX1	Apurinic/apyrimidinic endodeoxyribonuclease 1
ASIC1a	Acid sensing ion channel 1a
AST	Adeno-to-squamous transdifferentiation
ATR	Ataxia telangiectasia and Rad3-related
ATP	Adenosine triphosphate
BAK1	BCL2 antagonist/killer1
BBB	Blood-brain barrier
BCRP/ABCG2	Breast cancer resistance protein
BLP	Bionic lipoprotein
BRCA1/2	Breast cancer gene1/2
CAFs	Cancer-associated fibroblasts
CAR	Chimeric antigen receptor
CDKs	Cyclin-dependent kinases
cfDNA	Cell-free DNA
CHD4	Chromodomain helicase DNA-binding protein 4
CHK1	Checkpoint kinase 1
CML	Chronic myeloid leukaemia
CML-BP	Blastic phase of chronic myeloid leukaemia
CNS	Central nervous system
CQ	Chloroquine
CRC	Colorectal cancer
CSCs	Cancer stem cells
ctDNA	Circulating tumour DNA
CTLA4	Cytotoxic T-lymphocyte-associated protein 4
CYP	Cytochrome P450
CYP3A4	CYP isoforms 3A4
DEHP	Di(2-ethylhexyl) phthalate
DNMT1/UHRF1	DNA methyltransferases1/ Ubiquitin-like containing PHD Ring Finger 1
Dox	Doxorubicin
Dox-Mel PL	Doxorubicin-melittin polymersome
DTEPs	Drug-tolerant expanded persisters
DTPs	Drug-tolerant persisters
ECM	Extracellular matrix
EGFL7	EGF such as domain multiple 7
EGF	Epidermal growth factor
EGFR	Epidermal growth factor receptor
Egr-1	Early growth response-1
EMT	Epithelial-mesenchymal transition
EOC	Epithelial ovarian cancer
ER+	Oestrogen receptor positive
5-FU	5-fluorouracil
FAM46A	Family with sequence similarity 46, member A
FGFR4	Fibroblast growth factor receptor 4
FGFR1	Fibroblast growth factor receptor 1
FLT3	FMS-related tyrosine kinase 3 receptor
FUT6	Fucosyltransferase VI
GDF-15	Growth differentiation factor-15
GPER	G-protein coupled oestrogen receptor
GR/OR	Gefitinib/osimertinib-resistant
GST	Glutathione S-transferase
H3K27ac	Histone H3 lysine 27 trimethylation acetylation
H3K27me3	Histone H3 lysine 27 trimethylation
HCC	Hepatocellular carcinoma
HGF	Hepatocyte growth factor
HER2/erbB2	Human epidermal growth factor receptor 2
HER2+	Human epidermal growth factor receptor 2 positive
HKDC1	Hexokinase domain containing protein-1
iANG-1	Intracellular angiopoietin-1
IFN	Interferons
IFN-γ	Interferon-gamma
IGFR	Insulin-like growth factor receptor
IGF-1R	Insulin-like growth factor 1 receptor
ISR	Integrated stress response
JAK/STAT	Janus kinase/signal transducers and activators of transcription
KDM5A	Lysine demethylase 5A
lncRNA	Long non-coding RNAs
lncRNA UCA1	LncRNA urothelial carcinoma-associated 1
LSD1/KDM1A	Lysine demethylase 1A
m6A	N6-methyladenosine
MAPK/ERK	Mitogen-activated protein kinase/extracellular signal-regulated kinase
MAPK1	Mitogen-activated protein kinase 1
MARM2.0	Second-generation MAPK Adaptive Resistance Model
MDR	Multiple drug resistant
MDSCs	Myeloid-derived suppressor cells
Mel	Melittin
miRNAs	MicroRNAs
MITF	Melanocyte inducing transcription factor
MRD	Minimal residual disease
MRP1/ABCC1	Multidrug resistance-associated protein 1
MSCs	Mesenchymal stromal cells
MUC17	Mucin 17
MVP	Major vault protein
ncRNAs	Non-coding RNAs
NEAT1	Nuclear paraspeckle assembly transcript 1
NF-κB	Nuclear factor kappa-light-chain-enhancer of activated B cells
NGFR	Nerve growth factor receptor
NGS	Next-generation sequencing
NK	Natural killer
NLGN3	Neuroligin-3
Nrf2	Nuclear factor erythroid 2–related factor 2
NSCLC	Non-small-cell lung cancer
PCR	Polymerase chain reaction
PCSK9	Proprotein convertase subtilisin/kexin type 9
PD-1	Programmed-cell death protein-1
PDGF	Platelet-derived growth factor
PDGFR	Platelet-derived growth factor receptor
PD-L1	Programmed death ligand 1
P-gp/MDR1/ABCB1	P-glycoprotein
pHe	Extracellular pH
pHi	Intracellular pH
PI3K/Akt/mTOR	Phosphoinositol-3-kinase/protein kinase B/mammalian target of rapamycin
PTKs	Protein tyrosine kinases
REG4	Regenerating gene 4
ROS	Reactive oxygen species
ROS1	Ros oncogene 1
RTKs	Receptor tyrosine kinases
rTMB	Relative tumour mutation burden
SNP	Single nucleotide polymorphism
SOD2	Superoxide dismutase 2
SOX2	SRY-Box transcription factor 2
TAMs	Tumour-associated macrophages
Tβ4	Thymosin 4
TFF3	Trefoil factor 3
TGF-β	Transforming growth factor-beta
TIS	Therapy-induced senescence
TKIs	Tyrosine kinase inhibitors
TME	Tumour microenvironment
TNBC	Triple negative breast cancer
Treg	Regulatory T
TYMS	Thymidylate synthase
UGT	Uridine diphosphoglucuronosyltransferase
Wnt	Wingless-related integration site
VEGF	Vascular endothelial growth factor
VEGFR	Vascular endothelial growth factor receptor
VESPA	Virtual Enrichment-based Signalling Protein-activity Analysis
